# Neurofilament Heavy Polypeptide Regulates the Akt-β-Catenin Pathway in Human Esophageal Squamous Cell Carcinoma

**DOI:** 10.1371/journal.pone.0009003

**Published:** 2010-02-03

**Authors:** Myoung Sook Kim, Xiaofei Chang, Cynthia LeBron, Jatin K. Nagpal, Juna Lee, Yiping Huang, Keishi Yamashita, Barry Trink, Edward A. Ratovitski, David Sidransky

**Affiliations:** 1 Head and Neck Cancer Research Division, Department of Otolaryngology, The Johns Hopkins University School of Medicine, Baltimore, Maryland, United States of America; 2 Department of Dermatology, The Johns Hopkins University School of Medicine, Baltimore, Maryland, United States of America; 3 Department of Surgery, Kitasato University Hospital, Sagamihara, Kanagawa, Japan; Texas A&M University, United States of America

## Abstract

Aerobic glycolysis and mitochondrial dysfunction are common features of aggressive cancer growth. We observed promoter methylation and loss of expression in neurofilament heavy polypeptide (NEFH) in a significant proportion of primary esophageal squamous cell carcinoma (ESCC) samples that were of a high tumor grade and advanced stage. RNA interference-mediated knockdown of NEFH accelerated ESCC cell growth in culture and increased tumorigenicity *in vivo*, whereas forced expression of NEFH significantly inhibited cell growth and colony formation. Loss of NEFH caused up-regulation of pyruvate kinase-M2 type and down-regulation of pyruvate dehydrogenase, via activation of the Akt/β-catenin pathway, resulting in enhanced aerobic glycolysis and mitochondrial dysfunction. The acceleration of glycolysis and mitochondrial dysfunction in NEFH-knockdown cells was suppressed in the absence of β-catenin expression, and was decreased by the treatment of 2-Deoxyglucose, a glycolytic inhibitor, or API-2, an Akt inhibitor. Loss of NEFH activates the Akt/β-catenin pathway and increases glycolysis and mitochondrial dysfunction. Cancer cells with methylated NEFH can be targeted for destruction with specific inhibitors of deregulated downstream pathways.

## Introduction

Esophageal squamous cell carcinoma (ESCC) is the eighth most common type of caner, accounting for more than 90% cases of esophageal cancer worldwide [1], [2]. Most patients with ESCC are diagnosed at an advanced stage, and metastasis to the regional lymph nodes occurs frequently [3]. The development of ESCC is associated with the accumulation of multiple genetic/epigenetic alterations, including the activation of oncogenes and/or the inactivation of tumor suppressor genes. Amplification and over-expression of cyclin D1 [4], alterations in the p53 and p16 pathways [5], [6], and mutations of p53 [7]–[9] and retinoblastoma [10] are involved in development of ESCC. In addition, hypermethylation of gene promoters and the corresponding loss of gene expression are now recognized as a hallmark of human cancer [11]. Most of the epigenetically silenced genes identified possess tumor suppressive activity [11]. Promoter methylation of MGMT [12] and RASSF1 [13] are involved in development of ESCC. We have focused on the discovery of additional tumor suppressor genes in ESCC inactivated by promoter methylation [14].

β-catenin is a multifunctional protein involved in cell-cell adhesion and signal transduction. In the Wnt signaling pathway, it regulates cellular differentiation and proliferation. APC protein targets β-catenin for destruction by interaction with β-catenin [15], which requires phosphorylation of β-catenin by Gsk3β on 3 serines and one threonine residue, all of which are encoded in exon 3 of the β-catenin gene [15], [16]. Deletion of the NH_2_ terminus or mutation of one or more of the exon 3 inhibits the ubiquitin-mediated degradation of β-catenin, increasing its availability as a transcriptional activator. In colorectal cancers [17] and several other tumors [18], [19], loss of control of intracellular β-catenin levels through mutation to either β-catenin or APC result in stabilization of β-catenin and accumulation of the protein in the cytoplasm. The increased concentration of β-catenin in the cytoplasm favors its binding to the T-cell factor (TCF) family of DNA-binding proteins [17], and it subsequently translocates to the nucleus where it induces transcription of specific genes which are involved in oncogenic transformation [20]–[30]. In the development of esophageal cancer, dysfunction of Wnt/β-catenin signaling has been implicated [26], however, in contrast to colorectal cancer, mutation in adenomatous polyposis coli (APC) or β-catenin gene is a rare event in esophageal cancer [27]-[30]. Increased expression and nuclear localization of β-catenin protein are reported in ESCC [19], [27], [31]–[33], suggesting that the accumulation of β-catenin in ESCC development does not result from the genetic alterations of either the β-catenin or the APC gene. Further mechanisms for nuclear and cytoplasmic β-catenin accumulation are likely to exist in esophageal carcinogenesis.

The neurofilament heavy polypeptide (NEFH, 200 kD) gene resides at chromosomal band 22q12.2 and was proposed as a DNA marker for presymptomatic diagnosis in neurofibromatosis type 2 (NF2) families [34], [35]. The NEFH encoding neurofilament heavy chain is one of the major components of the neuronal cytoskeleton neurofilaments [36]. The role of NEFH has been studied extensively in motor neurons that require a high level of mitochondrial activity and harbor increased neurofilament content compared to other neuronal groups [37]. Recently, an interaction between neurofilaments and brain mitochondria was found to be mediated by NEFH, and the binding was dependent on the potential of the mitochondrial membranes [38]. Interestingly, both NEFH and β-catenin are constituent proteins in the postsynaptic density (PSD) in the mammalian central nervous system [39], suggesting a cooperative function of NEFH and β-catenin in the PSD.

Loss or down-regulation of NEFH has been mostly reported in human autonomic nerve tumors or central neurocytomas [40]–[43]. In addition, absent or diminished NEFH expression in human prostate cancer [44], clear-cell epithelioid tumor [45], and small cell lung carcinoma [46] has been observed. Interestingly, over-expression of NEFH disrupted normal cell structure and function, and induced cell death [47]. Fibroblasts cells expressing high amounts of NEFH were strongly misshapen, multinucleated, and had many inclusions, typical changes seen in neuropathies [47]. These results suggest that NEFH is needed for maintaining normal cell integrity and diminished NEFH levels are seen in common cancers. However, precise function of NEFH in the human cancer development remains to be investigated.

Here, we report NEFH as a novel tumor suppressor in ESCC. Its expression is down-regulated by hypermethylation of the CpG island in the promoter, and loss of NEFH increases glycolysis and mitochondrial dysfunction through activation of the Akt/β-catenin pathway. Epigenetic silencing of the NEFH gene in ESCC seems to be responsible for increased β-catenin expression, leading to activation of β-catenin/TCF-dependent transcription and key downstream effectors, leading to ESCC progression.

## Results

### NEFH Promoter Is Methylated and Its Expression Is Down-Regulated in ESCC

**Figure 1:**
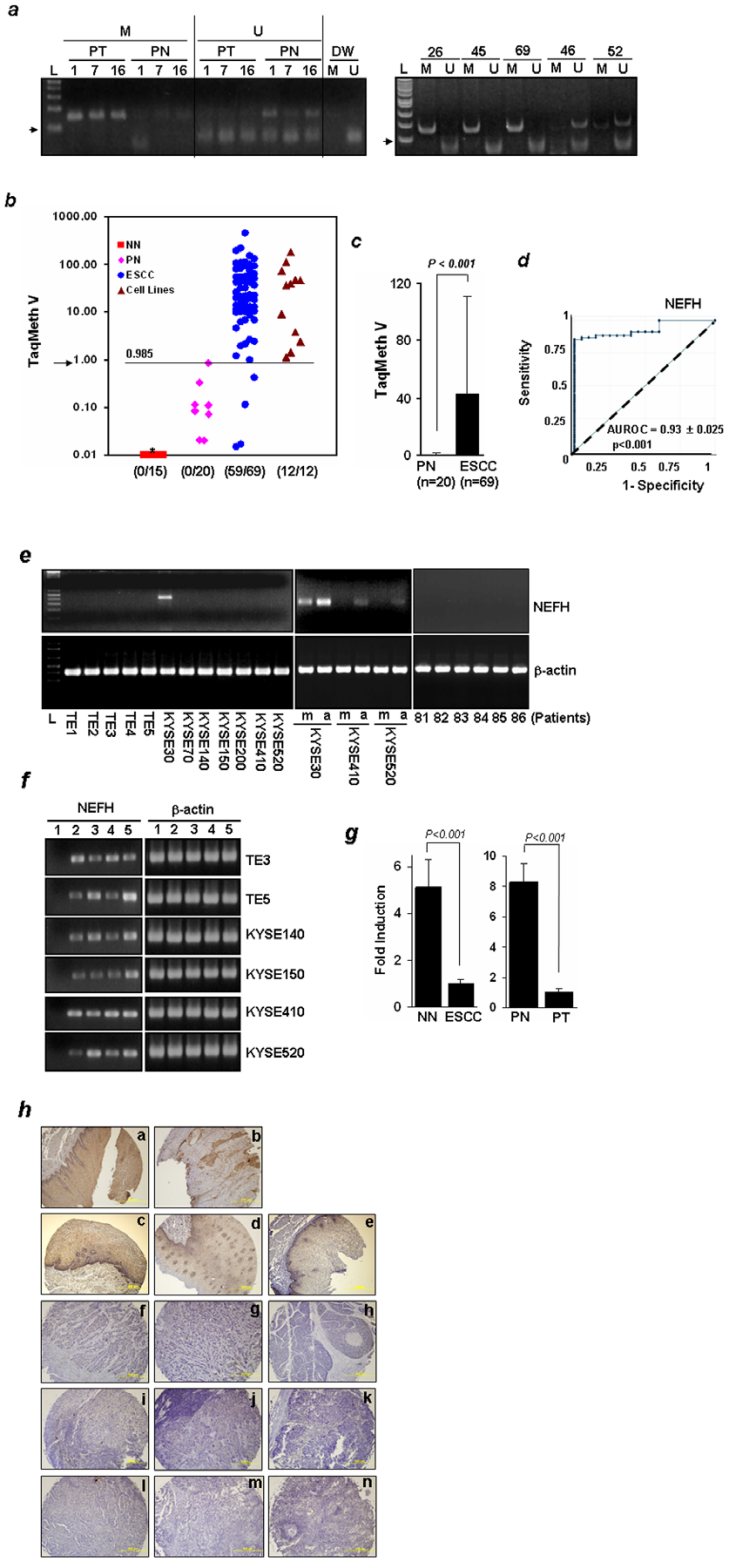
Analysis of NEFH methylation and expression in ESCC. ***a*** **.** Methylation status of the NEFH gene was examined by MSP with methylation (M)- and unmethylation (U)-specific primers. PCR products were run on 4% agarose gels pre-stained with ethidium bromide. PN, paired normal esophageal tissues from ESCC patients; PT, paired ESCC. Patients numbers are indicated. Corresponding normal tissues from patients 26, 45, 69, 46, and 53 were not available. Distilled water (DW) was used as a negative PCR control. L, 1 Kb Plus DNA ladder. Arrows, 100 bp. ***b***, Scatter plots of methylation values of NEFH in tissues and cell lines. The overall methylation value (TaqMan methylation values, TaqMeth V) is described in Materials and Methods. Arrow, the optimal cut-off value for NEFH. *, Samples with a ratio equal to zero could not be plotted correctly on a log scale, so are presented here as 0.01. All assays were performed in duplicate format, and experiments were repeated twice. Data showed reproducible and concordant results in triplicate. Note, six ESCC tissues (8.7%, 6/69) harbored a 3-bp deletion that removes one amino acid (Ser45) in one of two *CTNNB1* alleles (data not shown). ***c***, Quantitative evaluation of NEFH methylation levels showed that the NEFH methylation level (TaqMeth V) was higher in ESCC (n = 69) than in PN (n = 20) (*P<0.001, Wilcoxon-Mann-Whitney* Test). ***d***, ROC conveys the accuracy in distinguishing ESCC from PN in terms of sensitivity and specificity. Area under ROC (AUROC) was over 0.93. Solid line, NEFH; dashed line, no discrimination. ***e***, Expression of NEFH in cell lines was examined by RT-PCR analysis. No basal expression of NEFH was seen in all ESCC cell lines except for KYSE30 (left) where baseline expression was seen despite promoter methylation. NEFH methylation correlated tightly with loss of gene expression in most of the ESCC cell lines. Silenced NEFH was reactivated after treatment with the demethylating agent, 5-Aza-dC, in 3 ESCC cell lines (KYSE30, KYSE410 and KYSE520) (middle). Expression of NEFH in cDNA from ESCC tissue was barely detected by RT-PCR (right). **β-actin** was used as a loading control. m, no treatment. a, 5-Aza-dC treatment. L, 1 Kb Plus DNA ladder. **f,** ESCC cell lines were treated with 5-Aza-dC and/or TSA, a histone deacetylase inhibitor. 1, no treatment; 2, 1 µM 5-Aza-dC; 3, 1 µM 5-Aza-dC + 300 nM TSA; 4, 5 µM 5-Aza-dC; 5, 5 µM 5-Aza-dC + 300 nM TSA. ***g***, Expression of NEFH was compared between a patient with ESCC and a patient without cancer (NN) (left), and in a pair of normal and tumor cDNA prepared from an ESCC patient (right) by real-time RT-PCR. Fold induction was calculated by comparing the ratios of mRNA expression of NEFH to an internal control gene, 18 s rRNA. Experiments were done in duplicate, and values indicate means ± SD. ***h***, IHC analysis of NEFH in esophagus cancer tissue array. a & b, PN and PT of an ESCC patient (Grade I); c, normal esophagus tissue; d, chronic esophagitis; e, hyperplasia; f, ESCC-Grade I; g, ESCC-Grade III; h, basaloid ESCC; i, metastatic ESCC-Grade I; j, metastatic ESCC-Grade II; k, metastatic ESCC-Grade III; l, adenocarcinoma-Grade II; m, adenocarcinoma-Grade III; n, metastatic adenocarcinoma-Grade II. a ∼ b were from ES241 and c ∼ n were from ES804.

After identifying NEFH as a candidate methylated gene in ESCC [14], we treated gDNA with bisulfite and re-sequenced the NEFH promoter in 12 ESCC cell lines and 20 pairs of primary ESCC (PT) with their corresponding normal esophageal tissues (PN). All 12 ESCC cell lines tested and 65% (13/20) of PT harbored NEFH promoter methylation, whereas no methylation was found in paired normal samples (PN) (0%) and two non-tumorigenic cell lines, HEK293 (Fig. S1). Methylation of the NEFH promoter was further confirmed by conventional methylation-specific PCR (MSP) in three randomly selected pairs of normal and tumor tissue samples. PCR-amplification with methylation-specific primers was clearly seen only in PT whereas amplification of non-methylated-DNA was seen only in PN, consistent with the results of bisulfite-sequencing (Fig. 1*a*, left). MSP analysis in primary ESCC tissues from five more patients demonstrated NEFH promoter methylation in 3 cases while the remaining two did not harbor methylation (Fig. 1*a*, right).

To quantify promoter methylation, real-time TaqMan-MSP analysis was performed with a probe targeted to the CpG island of NEFH. Forty-nine cases of primary ESCC and 15 normal esophageal epithelial tissues from non-cancer patients (NN) were included to compare methylation levels between cancer and non-cancer patients. The distribution of methylation values in each group of samples is shown in Figure 1*b*. The overall TaqMan methylation value (TaqMeth V) detected in primary ESCC (42.84±68.13, mean ± SD) was significantly higher than that in normal tissues (0.08±1.63, mean ± SD) (*P*<*0.001*) (Fig. 1*c*). Testing methylation of NEFH resulted in a highly discriminative receiver–operator characteristic (ROC) curve profile, clearly distinguishing ESCC from PN (Fig. 1*d*). The optimal cut-off (value, 0.985) was calculated from the ROC analysis in order to maximize sensitivity and specificity. No NN nor PN samples exhibited a value over 0.985, yielding 100% specificity, while 85.5% (59/69) of primary ESCC tissues displayed NEFH promoter methylation (*P<0.001*, ESCC *vs.* PN, *Fisher's exact* test). No correlation between clinical features and NEFH methylation was found.

If functionally relevant, promoter methylation should correlate with decreased expression or silencing of the gene. To examine the transcriptional levels of NEFH, RT-PCR was performed using primers specific for NEFH cDNA. NEFH expression was hardly detectable in most of the ESCC cell lines except for KYSE30 (Fig. 1*e*, left). Moreover, NEFH expression was reactivated by 5-Aza-dC in all ESCC cell lines, and the reactivation was stronger in TE5, KYSE140, KYSE150, and KYSE520 but decreased or minimally different in TE3 or KYSE410 when both 5-Aza-dC and 300 nM TSA were used (Fig. 1*f* and data not shown). Although NEFH was expressed in KYSE30 at baseline, it was further increased by 5-Aza-dC (Fig. 1*e*, middle), indicating that its expression was at least partially suppressed by methylation. These results suggest that promoter hypermethylation of NEFH is one of the causal factors of loss of NEFH expression.

We then performed RT-PCR and real time RT-PCR in cDNA prepared from tumor and normal tissues, and found that NEFH was significantly down-regulated in ESCC (Fig. 1*e*, right and Fig. 1*g*). To investigate protein expression of NEFH, we performed immunohistochemical (IHC) staining using ESCC tissue arrays. Among 26 pairs of ESCC (PT) with matched normal adjacent tissues (PN) analyzed, decreased NEFH expression was observed in 10 cases (38.5%) (Table S1 and S2). NEFH expression was detected in 24 out of 26 cases (92.3%) of non-malignant esophageal tissues (normal, inflammation and hyperplasia), whereas absent or faint expression of NEFH was observed in 35 out of 47 cases (74.5%) of neoplastic tissues (Table 1 and Table S3). When compared to normal esophagus tissue, the negative expression of NEFH in adenocarcinoma (75%, 15/20) (*P<0.001*), squamous cell carcinoma (66.6%, 12/18) (*P = 0.002*), and metastatic cancer (88.8%, 8/9) (*P<0.001*) was statistically significant but this was not the case in hyperplasia (20%, 2/10) (*P = 0.477*) (Fig. 1*h*).

**Table 1:** Immunohistochemical analysis of NEFH in esophageal cancer tissue microarray with normal tissue controls (ES804).

			NEFH (+)	%	*P value*
Non-malignant	Total		24/26	92.31	
	Normal		8/8	100.00	
	Chronic esophagitis		8/8	100.00	
					
	Hyperplasia	Subtotal	8/10	20.00	*0.477*
		*Hyperplasia of epithelium*	2/2	100.00	
		*Atypical hyperplasia*	6/8	75.00	
Malignant	Total		12/47	25.53	
	Adenocarcinoma	Subtotal	5/20	25.00	*<0.001* *
		*II*	4/10	20.00	
		*II-III, III*	1/8	12.50	
		*Adenosquamous carcinoma*	0/1	0.00	
		*Basaloid squamous cell carcinoma*	0/1	0.00	
	Squamous cell carcinoma	Subtotal	6/18	33.33	*0.002* *
		*I*	4/11	36.36	
		*I-II, II*	1/4	25.00	
		*III*	1/3	33.33	
	Metastatic cancer	Subtotal	1/9	11.11	*<0.001* *
		Metastatic adenocarcinoma	1/3	33.33	
		Metastatic squamous cell carcinoma	0/6	0.00	

Expression level was indicated as -, absent or faint expression; +, moderate expression; ++, expression; +++, strong expression.

NEFH positivity (+) was counted in samples with over moderate expression.

*P values* from *Fisher's exact* test performed in normal vs. hyperplasia, adenocarcinoma, squamous cell carcinoma, or metastatic cancer.

* P<0.05 considered significant.

### Knockdown of NEFH Increases Tumor Growth

**Figure 2:**
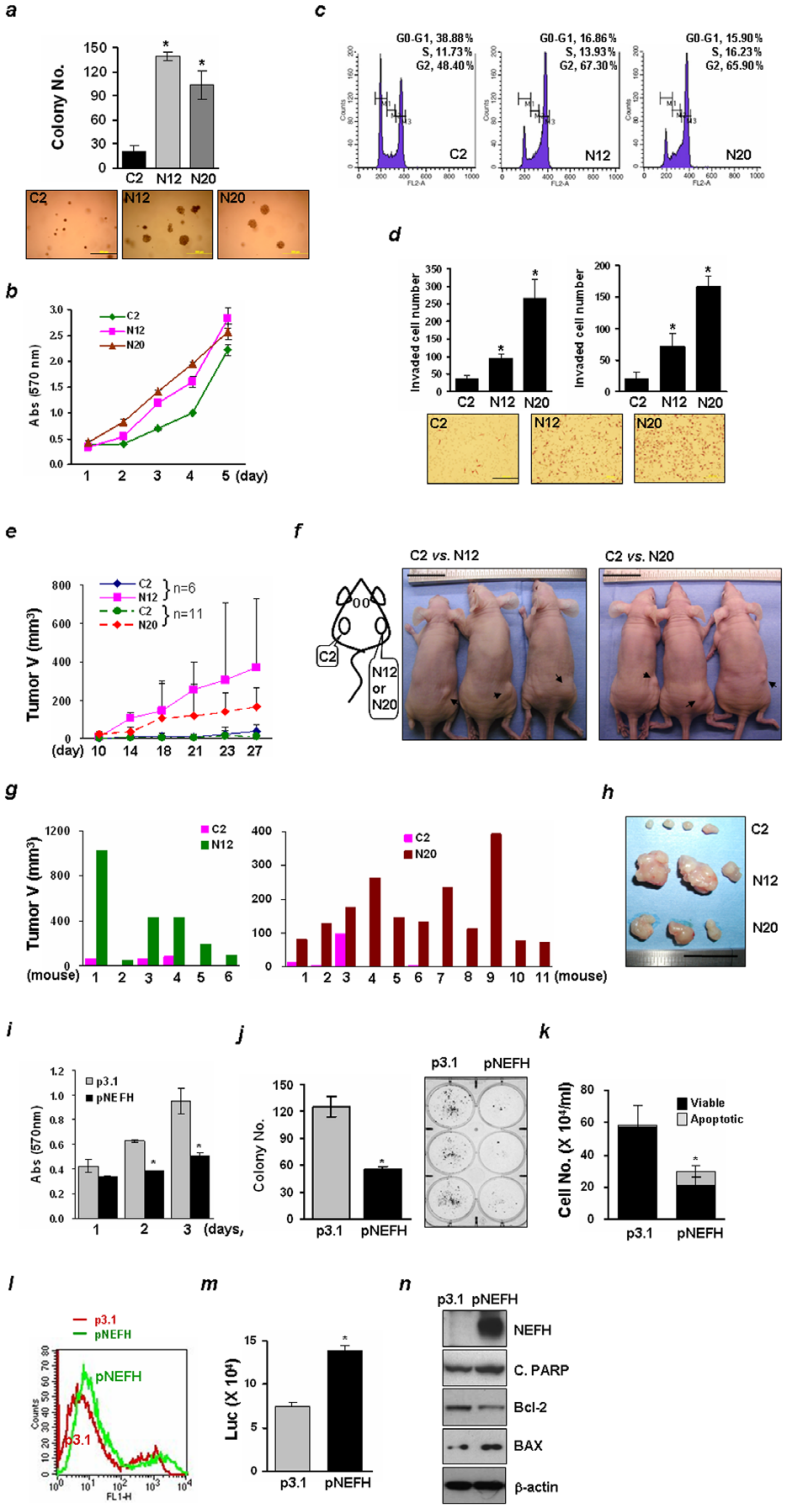
Down-regulation of NEFH increases tumor growth. ***a***, Soft agar assays were performed with the stable cell lines. Colonies were counted and photographed under a microscope after two weeks of cell culture. Scale bar, 500 µm. *, *P<0.05* considered significant (*T*-test). ***b***, Cellular growth was evaluated by the MTT cell growth assay for 5 days. Data are expressed as absorbance at 570 nm and experiments were repeated twice in triplicate. ***c***, Representative images of cell cycle analysis. After serum starvation for 24 hrs, cells were released in 10% serum-medium for 24 hrs for cell cycle transition. Cells were fixed, incubated in propidium iodide/RNase A solution, and analyzed by flow cytometry. Two independent experiments were done and similar results were observed. ***d***, Cells were incubated in 10% serum medium (upper left) or in 1% serum with HGF (100 ng/ml, upper right) for 16 hrs, and the *in vitro* cell invasion assay was performed. HGF promotes cell migration of human ESCC [70]. After fixation and staining, invading cells were counted at 40 X magnification (upper, left) or 100 X magnification (right). Cells were photographed at 40 X magnification (lower). Scale bar, 200 µm. Cell growth for 16 hrs determined by MTT assay was not significant (data not shown). Two independent experiments were done in triplicate, and values indicate means ± SD. ***e***, Knockdown of NEFH in tumorigenicity of KYSE30 cells. C2 cells were injected on the left and N12 or N20 cells were on right flank of each of 6-week-old nude mice, and the time course of tumor growth was measured twice a week for 4 weeks as described in Material and Methods. Each point represents the mean volume ± S.D. of tumor volumes of mice in each group. The variance of the tumor volume in mice injected with N20 cells was smaller than in mice injected with N12 cells, suggesting that the N12 cells were probably a mixed population of clones with different levels of NEFH knockdown. ***f***, At day 27 after injection, mice were sacrificed and pictures were taken. Scale bar, 2.5 cm. Arrows indicate tumor mass in individual mice. At day of 27, tumor mass was dissected from both flanks and tumor volume was measured **(** ***g*** **)**. ***h***, Representative pictures for tumor mass. Scale bar, 2.5 cm. ***i***, the MTT assay was performed in KYSE140 cell lines transiently transfected with an expression vector carrying NEFH full-length cDNA (pcDNA3.1-NEFH, pNEFH) or a mock vector pcDNA3.1 (p3.1) as a control. Data are expressed as absorbance at 570 nm and two independent experiments were done in triplicate. Values indicate means ± SD. *, *P<0.05* in *T-*test. ***j***, Colony focus assays were performed after transfection with NEFH in KYSE140. Cells were incubated in the presence of G418 (125 µg/ml) for two weeks and stained with 0.4% crystal violet solution (MeOH/Acetic acid, 3∶1). After air-drying, colonies were photographed under a microscope. Values are expressed as means ± SD and are derived from experiments done in triplicate. ***k***, The trypan blue dye exclusion assay was performed to determine the number of viable or apoptotic cells present in a cell suspension. Live cells that had intact cytoplasms (white) and apoptotic cells that did not (blue) were counted, and data are presented as cell number per ml of cell suspension. Experiments were repeated twice in triplicate. ***l***, Apoptosis was quantified using the Annexin V analysis. Labeled cells were detected by Flow cytometry analysis. Cell distribution of NEFH-expressing cells was shifted toward increased apoptosis. ***m***, Caspase-3/7 activity was determined using luminescence (Luc)-based Caspase assay system. Cells were incubated for 72 hrs after transfection. Experiments were repeated twice in triplicate. ***n***, Western blotting of whole cell lysates extracted from KYSE140 cells 48 hrs after transfection. C.PARP, cleaved PARP. β-actin was used as a loading control.

Since the NEFH promoter was specifically methylated in tumor tissues and its expression was down-regulated in ESCC, NEFH might function as a tumor suppressor in esophageal cancer. In order to test this hypothesis, we first examined whether knockdown of NEFH increased cell growth after establishing NEFH shRNA stable clones in KYSE30 cells (Fig. S2*a*–S2*c*). A significant increase in clonogenic cell growth was observed in cells with diminished NEFH expression (N12 and N20) (Fig. 2*a*). Increased cell proliferation was also observed in N12 and N20 cells compared to control cells (C2) (Fig. 2*b*). Because cell proliferation is closely linked to progression of the cell cycle, cell cycle analysis was performed by flow cytometry. The population of N12 and N20 cells residing in the G_0_/G_1_ phase yet decreased in the G_2_ phase compared to C2 cells (Fig. 2*c*), indicating that cell cycle progression through G_0_/G_1_ and then a block at G_2_ occurred from loss of NEFH. In addition, *in vitro* invasive activity was increased in N12 and N20 cells (Fig. 2*d*). When cells were incubated in 10% serum medium, the number of cells passing into the chamber increased about 2.5- and 7-fold in N12 and N20 cells respectively, compared to C2 cells (Fig. 2*d*, left). Under HGF treatment, the invasive activity of N12 and N20 cells increased about 3.5- and 8-fold, respectively, compared to C2 cells (Fig. 2*d*, right).

To examine the loss of NEFH *in vivo*, cells were subcutaneously injected into the flanks of 6-week-old nude mice. Tumor development was observed 10 days after injection in both flanks injected with control or either N12 (n = 6) or N20 cells (n = 11). A marked increase of tumor volume was observed in mice injected with NEFH-knockdown cells (Fig. 2*e–h*). Tumor volume in the mice injected with C2 cells did not change significantly for 4 weeks after injection. IHC analysis confirmed decreased expression of NEFH in N12- or N20 cells-grafted tumors (Fig. S2 *d*). The expression of proliferating cell nuclear antigen (PCNA), a marker for cell proliferation, increased in N12 and N20 cells.

To examine the effects of increased NEFH expression on the growth of esophageal cancer cells, the KYSE140 cell line was selected due to its barely detectable basal expression of the NEFH gene (Fig. 1*e*). KYSE140 cells with a higher level of NEFH did not survive for longer than three weeks (data not shown), so transient transfection was performed for NEFH gene delivery. We first performed the MTT assay to compare cell growth with or without expression of NEFH. The growth in cells expressing NEFH decreased to 55% of control cells that exponentially grew for 3 days of incubation, (Fig. 2*i*) (*P*<0.05). We then performed the colony focus assay. In control cells, KYSE140 exhibited strong colony-forming ability with multiple colonies (125±11.24 colonies) (Fig. 2*j*). However, in pNEFH-transfected cells, a marked decrease in colony numbers was observed (55.33±6.43 colonies, 44% of control) (*P*<0.001). These results indicate that forced expression of NEFH suppresses cell growth.

To investigate whether NEFH had apoptotic activity, the population of live and dead cells were determined by the trypan blue exclusion assay. The population of non-viable, apoptotic cells having blue cytoplasm was less than 2% in control cells, whereas the population of non-viable cells in pNEFH-transfected cells increased to 27% (Fig. 2*k*). The apoptotic cell population was further assessed by flow cytometry after staining cells with Annexin V-FITC and 7-AAD, based on the cell population in the top right (late stage of apoptosis) and the bottom right quadrants (early stage of apoptosis). A 15% increase in apoptotic cells was observed in pNEFH-transfected cells compared to control with a distribution shift toward increased apoptosis (Fig. 2*l*). Forced expression of NEFH caused a 2-fold increase in the caspase−3/7 activity (Fig. 2*m*), but did not increase the caspase-9 activity (data not shown). Western blot analysis showed increased levels of cleaved PARP (C.PARP) and BAX, but decreased level of Bcl-2 in NEFH expressing cells (Fig. 2*n*), indicating that the mitochondria-mediated cell death pathway is activated by NEFH.

### NEFH-Knockdown Stimulates β-Catenin-TCF/Lef Signaling

**Figure 3:**
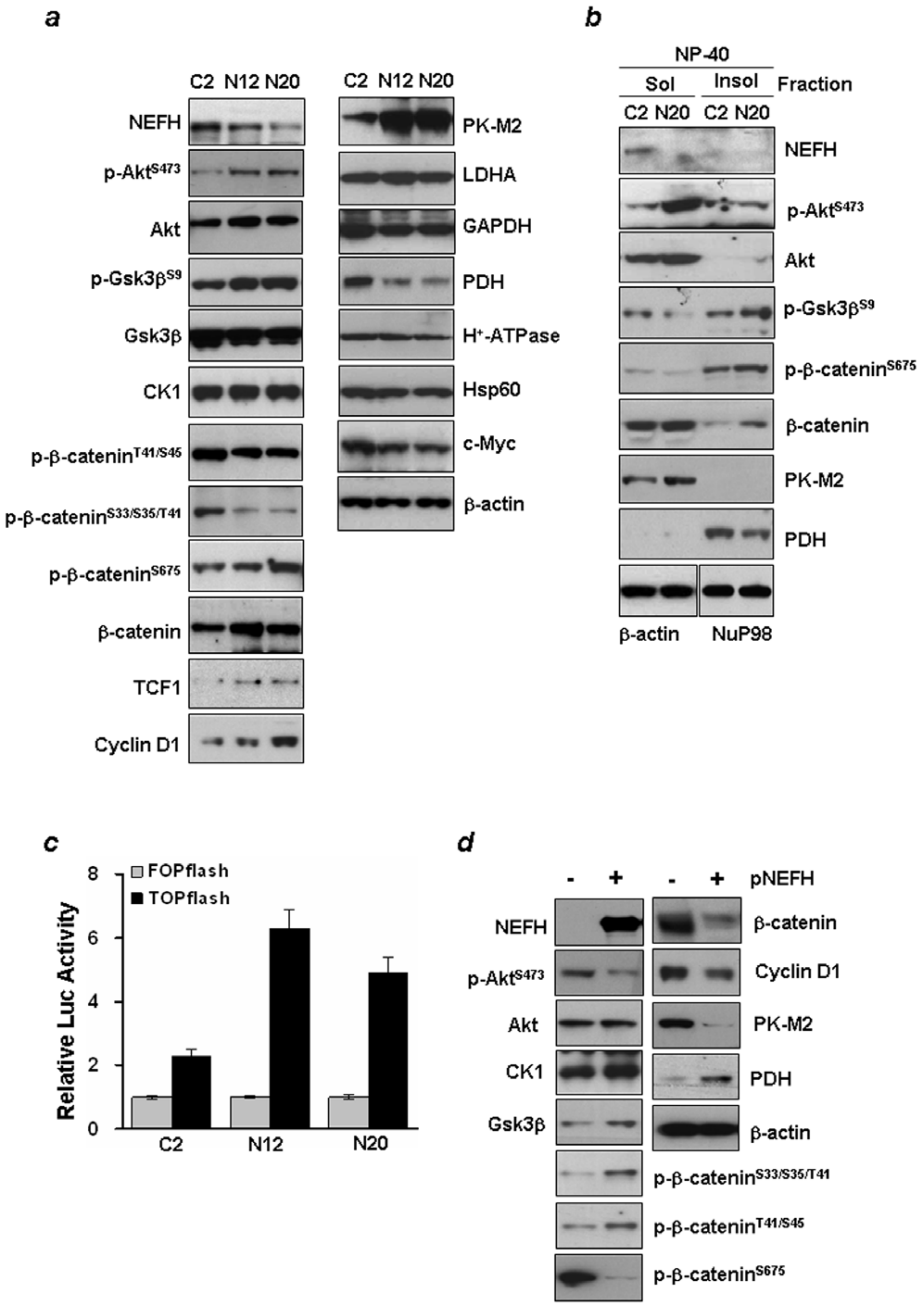
NEFH-knockdown stimulates β-catenin-TCF/Lef signaling. ***a***, Total cell lysates extracted from C2, N12 and N20 cells were run on 4–12% polyacrylamide gel and transferred onto nitrocellulose membrane. Membranes were then probed for expression of factors mediating β-catenin signaling and down-stream target genes (left), and proteins functioning in glycolytic pathways and in mitochondrial function using individual specific antibodies (right). No mutation was found in exon 3 of β-catenin in all 12 ESCC cell lines tested (data not shown), indicating that Gsk3β binding to β-catenin is intact in these cell lines. ***b***, Expression of proteins in NP-40-soluble (cytosolic) and -insoluble (nuclear) fractions. β-actin and nuclear protein 98 (NuP98) serve as loading controls for each fraction. ***c***, TOPflash reporter activity. C2, N12 and N20 cells were transfected with either TOPflash or FOPflash reporter plasmid. β-Catenin-TCF/Lef-mediated transcription was determined by the TOPflash luciferase activity. FOPflash contained mutant TCF/LEF-1-binding sites and was used as control. Reporter activities were normalized to internal control *Renilla*. Two independent experiments were done in triplicate, and values indicate means ± SD. ***d***, Western blotting of whole cell lysates extracted from KYSE140 cells 48 hrs after transfection.

Previous studies have shown that β-catenin-TCF/Lef signaling is aberrant in esophageal carcinomas [48]. Phosphorylated Akt triggers a network that positively regulates G_1_/S cell cycle progression through inactivation of Gsk3β [49], direct phosphorylation of β-catenin at Ser552, which enhances β-catenin nuclear accumulation [50] and increased β-catenin-TCF/Lef transcriptional activity [49]. Therefore, we investigated whether the expression of Akt and its downstream effectors was affected by NEFH down-regulation. Increased Gsk3β phosphorylation and decreased total Gsk3β level were observed in N12 and N20 compared to C2 cells (Fig. 3*a*, *left*). The slight increase of total Akt was due to increased Akt1 and Akt2 in N12 and N20 cells, respectively (Fig. S3*a*–S3*c*).

Both Gsk3β and casein kinase-1 (CK1) phosphorylate β-catenin at Ser33/Ser37/Thr41 and at Thr41/Ser45, respectively, leading to the degradation of β-catenin through the ubiquitin pathway [51], [52]. In contrast, protein kinase A (PKA) phosphorylates β-catenin at Ser675 to stabilize β-catenin, resulting in increased cytoplasmic and nuclear β-catenin levels [53]. In NEFH down-regulated cells, phosphorylation of β-catenin at Ser33/Ser37/Thr41 and at Thr41/Ser45 decreased with a concomitant increase of total β-catenin. In contrast, phosphorylation of β-catenin at Ser675 increased in N12 and N20 cells.

Activation of the Akt oncogene is in part responsible for the bioenergetic shift to dependence on glycolytic metabolism characteristic of most malignant cells [54]. Akt also plays a vital role in the maintenance of mitochondrial membrane integrity to prevent the induction of apoptosis [55], [56]. We thus further elucidated expression level of proteins that play roles in the glycolytic pathway and in mitochondrial function in NEFH-diminished cells. M2-type pyruvate kinase (PK-M2) is a key glycolytic enzyme, and pyruvate dehydrogenase (PDH) is a TCA cycle enzyme converting pyruvate to acetyl-CoA in mitochondria. PK-M2 and PDH are two of the enzymes that play a key role in increasing glycolysis and lactate production. The protein level of PK-M2 increased in N12 and N20 compared to C2 cells, whereas PDH decreased (Fig. 3*a*, right). A slight decrease of glyceraldehyde 3-phosphate dehydrogenase (GAPDH) was also observed in N12 and N20 cells, but little difference was observed in the expression of lactate dehydrogenase-A (LDHA), Hsp60, a mitochondrial matrix protein, or mitochondrial H^+^-ATP synthase (β-F1-ATPase). Interestingly, c-Myc expression, which increases mitochondrial biogenesis and cellular respiration [57], [58], was down-regulated after decreasing NEFH expression. Similar results were observed in HEK293 cells transfected with NEFH siRNA (Fig. S3 *d*). In addition, to determine whether the altered level of β-catenin phosphorylation affects the subcellular localization of β-catenin, NP-40 soluble (cytosol) and insoluble (nucleus) fractions were extracted from C2 and N20 cells. The level of β-catenin increased in the nuclear fraction of N20 cells (Fig. 3*b*), indicating increased stability of β-catenin in cells with loss of NEFH.

The luciferase reporters, TOPflash and FOPflash, which have either wild-type (TOP) or mutated (FOP) binding sites for the β-catenin-TCF4/Lef complex, were used to characterize the transcriptional activity of the β-catenin-TCF/Lef complex. These reporter constructs were transfected into C2, N12 and N20 cells, and the luciferase activity was determined. In C2 cells, TOPflash activity was 2-fold higher than FOPflash activity, whereas a 6- and 5-fold increase of TOPflash activity was detected in N12 and N20 cells, respectively (Fig. 3*c*), indicating that NEFH loss increases the transcriptional activity of the β-catenin-TCF/Lef complex. To investigate whether down-regulation of NEFH alters β-catenin-TCF/Lef-dependent transcription, the expression levels of cyclin D1, TCF1, and MMP-7 were examined. We found that the transcriptional levels of β-catenin, cyclin D1 and MMP-7 were elevated significantly in N20 compared to C2 cells (Fig. S3 *e*), indicating that stimulation of β-catenin-TCF/Lef signaling by NEFH down-regulation results in up-regulation of key target genes.

We also tested protein expression in control and KYSE140 cells expressing NEFH. While the level of total Akt was similar in the two cell types, phosphorylated Akt was decreased by NEFH (Fig. 3*d*). The levels of phospho-β-catenin^Ser675^ and of total β-catenin in both total cell lysates (Fig. 3*d*) and nuclear fraction were decreased by NEFH (data not shown). In contrast, the expression of Gsk3β, and phosphorylation of β-catenin^Ser33/Ser37/Thr41^ and β-catenin^Thr41/Ser45^ were increased by NEFH. The expression of cyclin D1 and MMP-7, downstream targets of β-catenin-TCF/Lef signaling, were down-regulated by NEFH. In addition, an increase of PDH and concomitant decrease of PK-M2 at the mRNA and protein level were observed in NEFH-expressing cells (Fig. 3*d* and Fig. S3 *f*). These results indicate that the expression of proteins involved in β-catenin-TCF/Lef signaling as well as levels of PK-M2 and PDH are altered by NEFH.

### Down-Regulation of NEFH Increases Mitochondrial Dysfunction and Glycolysis

Preservation of cellular survival requires association of a series of cellular survival pathways which are linked to the activation of Wnt signaling and Akt for the maintenance of the mitochondrial membrane potential. Akt has been coined the “Warburg kinase” and increased aerobic glycolysis is a common abnormality of human cancer [59]. Mitochondrial membrane potential (Δψm) is known to be the driving force of mitochondrial ATP synthesis, and a decrease in Δψm contributes to the acquisition of a more invasive cellular phenotype with reduced chemosensitivity [60]. To determine changes in Δψm by NEFH down-regulation, intracellular mitochondria were stained with the membrane potential sensitive dye, JC-1, and fluorescence imaging was performed by flow cytometry. The distribution of J-aggregates (FL2-H channel, Red Flu) in N20 cells decreased compared to C2 cells whereas that of the JC-1 mononer (FL1-H channel, Green Flu) increased, indicating decreased Δψm in N20 cells (Fig. S4*d* and S4*e*). The population of cells that resided at the top right (UR) and the bottom right quadrant (LR) was compared in Quadrant statistics. Cell population at the LR increased in N20 (2.34%) compared to C2 cells (0.46%). These results indicate disruption of the mitochondrial membrane potential by down-regulation of NEFH.

**Figure 4:**
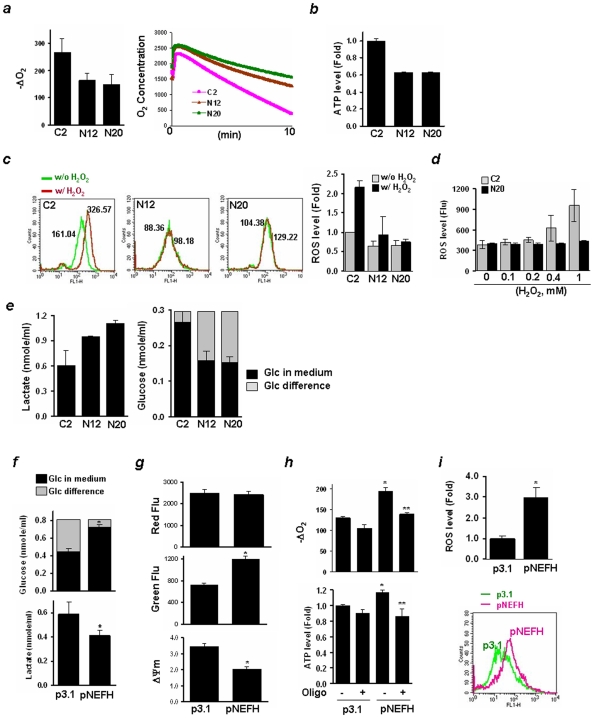
Increase of mitochondrial dysfunction and glycolysis by decreased NEFH. ***a***, Cells were incubated with a Clark-type electrode to measure O_2_ concentration as a function of time (right) and to calculate rates of O_2_ consumption (-ΔO_2_, nmol/min/10^7^) (left). ***b***, ATP level was measured using an equal number of cells (nmol/ml/10^6^), and data are presented as relative fold compared to control. ***c***, Representative results of ROS imaging with fluorescent intensity in each cell line (three in the left). The ROS level in cells was measured using DCDHF-DA fluorescent dye, and analyzed by flow cytometry. Cells were treated with H_2_O_2_ (100 µM) in serum-free medium for 2 hrs, and ROS levels were immediately measured without recovery. Relative fold values were calculated, and experiments were repeated twice in triplicate. Values indicate means ± SD (far right). ***d***, Cells in culture were exposed to different concentrations of H_2_O_2_ (0 ∼ 1 mM), and ROS levels were measured after 24 hrs of recovery in complete growth medium. Cells were analyzed using a fluorescence microplate reader and fluorescence was acquired at 495/525 (ex/em). ***e***, Lactate production (left) and glucose concentration (right) were measured in cell culture medium and the MTT assay was performed for verifying equal number of cells, and no significant difference was found (data not shown). ***f***, Glucose concentration (upper) and lactate production (lower) were measured in cell culture medium 24 hrs after transfection of pNEFH or p3.1 vector into KYSE140 cells. ***g***, Δψm was determined after staining cells with a membrane potential sensitive dye, JC-1. Fluorescence intensity was acquired by reading cells in a fluorescence microplate reader. Values are expressed as means ± SD, and experiments were done in triplicate. ***h***, O_2_ consumption (upper) and ATP synthesis (lower) were increased by NEFH expression in KYSE140 cells. *, *P<0.05* compared to KYSE140 cells transfected with p3.1; **, *P<0.05* compared to cells transfected with pNEFH without treatment of oligomycin (2.5 µM), an inhibitor of mitochondrial H^+^-ATPase. ***i***, ROS levels were measured using DCDHF-DA fluorescent dye, and analyzed by flow cytometry. Relative fold values were calculated and indicated as means ± SD (upper). Representative results of ROS imaging with fluorescent intensity (lower).

A metabolic switch from mitochondria-based, oxygen-dependent ATP production (oxidative phosphorylation) to aerobic glycolysis leads to oxygen- and mitochondria-independent ATP generation, which is a hallmark of aggressive cancer growth [38]. To determine whether the reduction in Δψm associated with NEFH loss reflects a reduction in respiration, the cellular O_2_ level was measured. As compared to C2 cells, we observed a decrease of cellular O_2_ consumption (-ΔO_2_) in N12 and N20 cells with a slow slope in O_2_ reduction as a function of time (Fig. 4*a*). Measurement of intracellular ATP content revealed reduced ATP synthesis in N12 and N20 cells (40% decrease) (Fig. 4*b*). Energy production by O_2_ consumption is required for cell survival, but dysfunctional mitochondria generate reactive oxygen species (ROS), resulting in cell dysfunction or death. The level of ROS was measured using a fluorescent dye, DCDHF-DA. The baseline ROS level in both N12 and N20 cells was about 65% of that in C2 cells (Fig. 4*c*). To investigate cell response to oxidative stress, cells were exposed to H_2_O_2_ in serum-free conditions for 2 hrs, and the ROS level was immediately measured. The distribution of ROS in C2 cells was shifted toward increased ROS by H_2_O_2_ treatment, whereas little change in N12 and N20 cells was observed. In separate experiments, cells were exposed to different concentrations of H_2_O_2_, and the ROS level was measured after 24 hrs of recovery. Consistent results were observed in N20 cells that exhibited reduced level of ROS compared to control (Fig. 4*d*). These results highlight that the cellular capacity to remove ROS increases when NEFH is lost, possibly resulting in increased resistance to oxidative stress (See Figure S4*a*–S4*c*).

The metabolic shift results in increased lactate production via cycling through the pentose phosphate pathway, and plays an important role in malignant transformation of cancer cells. We thus collected growth medium from cell culture for examination of lactate and glucose levels. An increase of over 40% in lactate production and glucose consumption was observed in N12 and N20 cells (Fig. 4*e*). Taken together, loss of NEFH expression resulted in decreased ROS production and increased aerobic glycolysis.

Since cellular respiration and glycolysis were deregulated by NEFH down-regulation, we examined the influence of NEFH expression on mitochondrial function and glycolysis. Lactate levels and glucose consumption were reduced by NEFH expression (Fig. 4*f*). In contrast, NEFH did not affect energized mitochondria (red fluorescence, J-aggregates), but increased the level of JC-1 monomers (green fluorescence); Δψm decreased in NEFH-transfected cells (Fig. 4*g*). The population of control cells in the bottom right quadrants (LR) was 4.15%, and increased to 9.15% in NEFH-transfected cells (data not shown). Significant increases of oxygen consumption and ATP generation were observed in NEFH-transfected cells (Fig. 4*h*), whereas oligomycin, an inhibitor of mitochondria H^+^-ATPase, inhibited the NEFH-increased oxygen consumption and ATP generation. Concomitantly, a 3-fold increase of ROS level was observed (Fig. 4*i*). These results indicate that NEFH regulates mitochondrial function, leading to alterations of cellular respiration and glycolysis.

### β-Catenin Is Required for the Inverse Regulation between PDH and PK-M2

**Figure 5:**
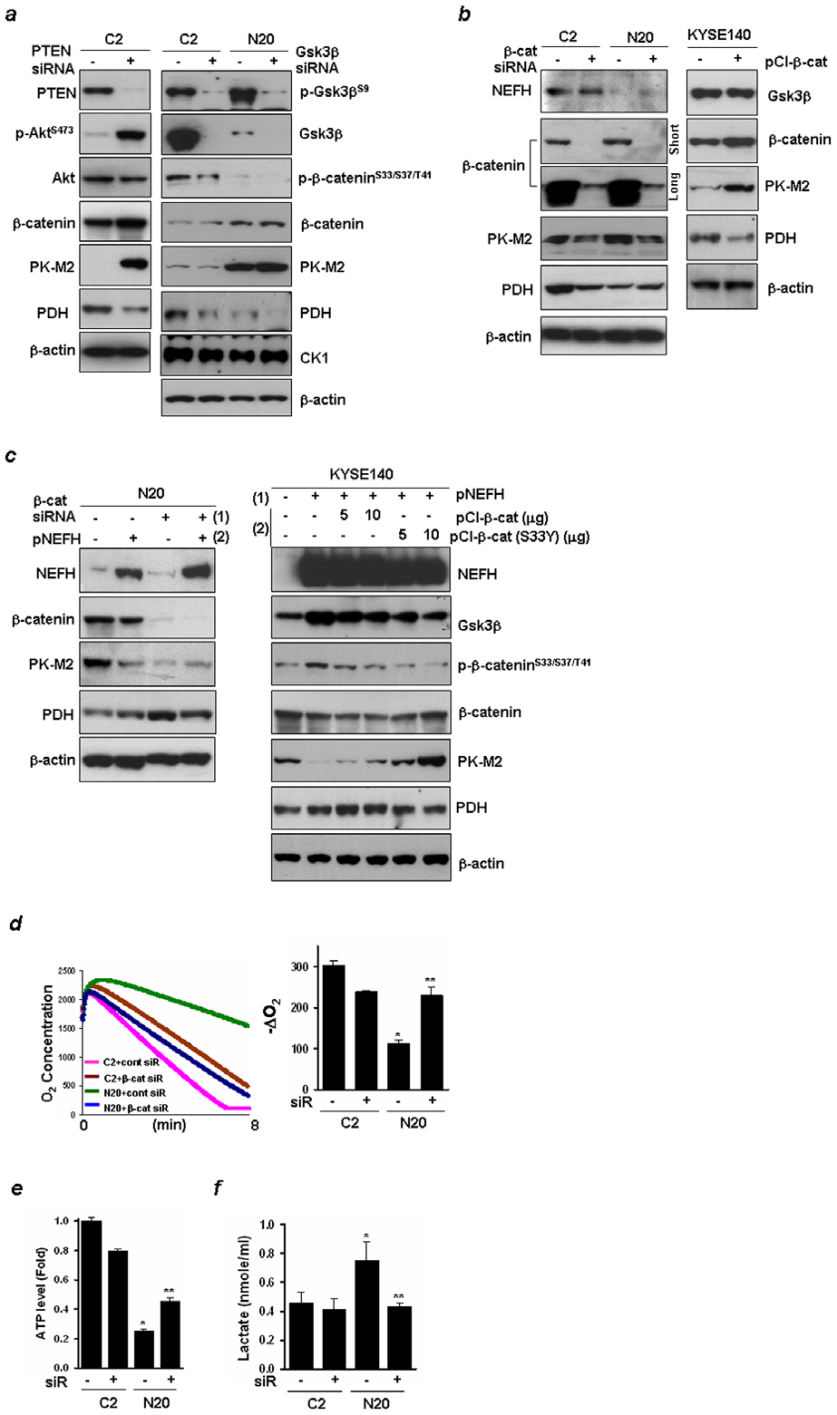
PK-M2 and PDH are regulated through the Akt-β-catenin pathway. ***a***, siRNA of PTEN (left), Gsk3β (right) or a non-targeting control was transfected into C2 and/or N20 cells and Western blot analysis was performed 48 hrs after transfection. β-actin was used a loading control. ***b***, Left, Cell were transfected with β-catenin or non-targeting control siRNA. For β-catenin detection, exposure time of the protein membrane on X-ray film was 5 sec (short) and 1 min (long) after extensive washing. Right, Expression levels of PK-M2 and PDH were compared in KYSE140 cells transfected with pCI-β-cat expressing wild-type β-catenin or mock plasmid. ***c***, Left, N20 cells were transfected with β-catenin or control siRNA and incubated for 48 hrs. Cells were then transfected again with pNEFH and p3.1 (10 µg each), incubated for another 48 hrs, and Western blot analysis was performed. Right, KYSE140 cells were transfected with pNEFH and incubated for 24 hrs, and then transfected again with pCI-β-cat or pCI-β-cat (S33Y) that expresses a constitutively active form of β-catenin, and incubated for another 48 hrs. ***d***, C2 and N20 cells were transfected with β-catenin or non-targeting control siRNA, and incubated for 48 hrs. After trypsinization, cells were incubated with a Clark-type electrode to measure O_2_ concentration as a function of time (left) and to calculate rates of O_2_ consumption (-ΔO_2_, nmol/min/10^7^) (right). Values are expressed as means ± SD and experiments were repeated twice in duplicate. *, *P<0.05* compared to C2 cells transfected with control siRNA. **, *P<0.05* compared to N20 cells transfected with control siRNA. ***e***, ATP level was measured using an equal number of cells (nmol/ml/10^6^), and data are presented as a relative fold value compared to control. ***f***, For the lactate assay, cells were incubated for 24 hrs after transfection, and cellular lactate level was measured in the cell culture medium.

Gsk3β is a key enzyme that phosphorylates β-catenin at NH_2_-terminal serine threonine residues. Gsk3β inactivation is mediated mainly by two pathways: 1) activation of growth factor receptors by EGF, platelet-derived growth factor or insulin, leading to activation of Akt, a protein kinase that phosphorylates and inactivates Gsk3β, 2) activation of the Wnt pathway leading to inhibition of Gsk3β. To investigate whether the Akt-β-catenin pathway is involved in the regulation of PK-M2 and PDH, each siRNA pool targeting PTEN, Gsk3β, β-catenin, or a non-targeting control was transfected into C2 and/or N20 cells, and western blot analysis was performed 48 hrs after transfection. As shown in Figure 5, more than 90% of each endogenous gene level was knocked down by the transfection. Activation of Akt with increased expression of β-catenin and PK-M2 was observed in C2 cells with PTEN gene knockdown (Fig. 5*a*, left). Gsk3β knockdown slightly increased the expression of PK-M2 in N20 cells, but not in C2 cells (Fig. 5*a*, right), indicating differences in knockdown efficiency of Gsk3β or the presence of Gsk3β-independent PK-M2 regulation in C2 cells. However, PDH expression was decreased by PTEN or Gsk3β siRNA transfection. In addition, β-catenin knockdown reversed the increased PK-M2 expression, and reversed the decreased level of PDH in N20 cells (Fig. 5*b*, left). Next, we transfected the pCI-β-cat plasmid expressing wt-β-catenin or mock control into KYSE140 cells and compared expression levels of PK-M2 and PDH. Over-expression of β-catenin increased PK-M2 but decreased PDH levels (Fig. 5*b*, right). These results indicate that PK-M2 and PDH are downstream effectors of the Akt-β-catenin pathway signaling in N20 cells.

To further elucidate whether β-catenin is required for the regulation on PK-M2 and PDH by NEFH, N20 cells were transfected with β-catenin or control siRNA and incubated for 48 hrs. Cells were then subsequently transfected again with pNEFH and p3.1, and incubated for a further 48 hrs. β-catenin levels remained down-regulated during 96 hrs of incubation after transfection (Fig. 5*c*, left). The up-regulated PDH by β-catenin knockdown was not further enhanced by the overexpression of NEFH, indicating that β-catenin is required for the inverse regulation of PK-M2 and PDH by NEFH. Moreover, KYSE140 cells were transfected with pNEFH or p3.1 and incubated for 24 hrs, and then transfected again with pCI-β-cat and incubated for a further 48 hrs. Even though β-catenin was forcibly expressed, its level was not higher than that of control, suggesting that the marked expression of NEFH possibly inhibits ectopic expression of β-catenin (Fig. 5*c*, right). No increase of PK-M2 and no decrease of PDH were observed in pCI-β-cat transfected cells. However, when KYSE140 cells were transfected with a β-catenin mutant (pCI-β-cat-S33Y) that has a defect in Gsk3β-dependent phosphorylation of β-catenin, NEFH could not suppress mt-β-catenin expression. Consequently, an increase of PK-M2 and a decrease of PDH were observed in the cells transfected with pCI-β-cat-S33Y. These results suggest that Gsk3β-dependent degradation of β-catenin contributes at least part to the tumor suppressive role of NEFH.

To examine whether β-catenin affects mitochondrial function, a siRNA pool targeting β-catenin or a non-targeting control was transfected into both C2 and N20 cells, and O_2_ consumption and ATP synthesis were examined 72 hrs after transfection. The reduced O_2_ consumption and ATP level in N20 cells were increased by β-catenin knockdown (Fig. 5*d and 5e*). However, Δψm (Fig. S5 *a*) and ROS level (data not shown) were not influenced by the β-catenin knockdown in both C2 and N20 cells. The increased lactate concentration in N20 cells returned to the level of C2 cells by β-catenin knockdown (Fig. 5*f*). In 24 hrs, cell viability was not significantly decreased by the low expression of β-catenin, but inhibition of cell growth by β-catenin knockdown was observed after 3 days of incubation (Fig. S5 *b*). These results suggest that β-catenin causes mitochondrial dysfunction in NEFH down-regulated cells by inverse regulation of PDH and PK-M2.

### NEFH Down-Regulation Increases Cellular Sensitivity to Glycolysis Inhibitors

To suppress the cellular glycolytic pathway, cells were treated with different concentrations of 2-deoxyglucose (2-DG), an inhibitor of glucose metabolism, or were exposed to glucose-free conditions for 24 hrs, and cell viability was examined. Glucose withdrawal did not make a significant difference in cell viability between C2 and N20 cells (50% of untreated control). However, reduction in the cell viability in N20 cells was significantly enhanced by 2-DG (Fig. S6 *a*).

**Figure 6:**
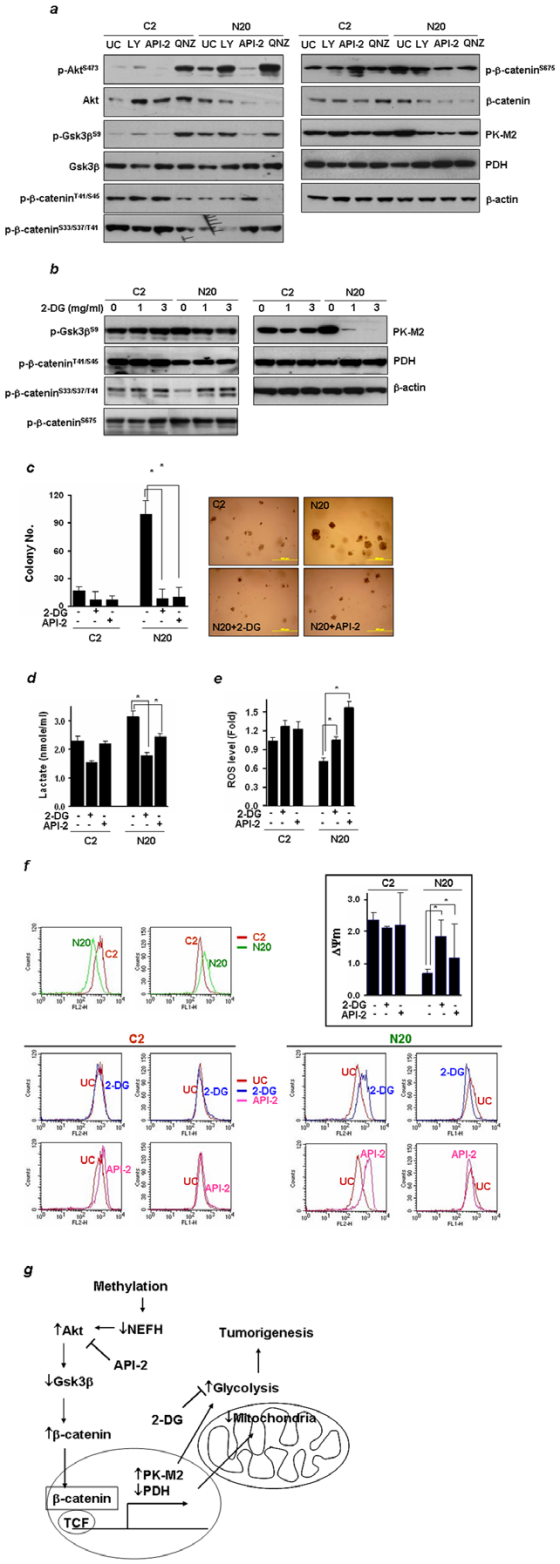
ESCC cells with loss of NEFH are susceptible to inhibition of glycolysis. ***a*** **&** ***b***, Expression levels of downstream effectors after treatment with 2-DG (1 mg/ml) or API-2 (10 µM) for 24 hrs. ***c***. Cells were incubated in the complete growth medium containing 2 mg/ml of D-glucose (RPMI 1640 medium) in the presence of 2-DG (1 mg/ml) or API-2 (10 µM). 2-DG or API-2 was treated three times a week for two weeks, and colonies were counted and photographed under a microscope. Values are expressed as means ± SD and experiments were repeated twice and done in triplicate. *, *P<0.05*. Scale bar, 500 µm. ***d***, Since cell death can skew the results, 2-DG and API-2 treatments were limited to 8 hrs, and lactate production was measured in the cell culture medium. Cell growth for 8 hrs determined by the MTT assay was not significant (Fig. S6 *e*). ***e***, Cellular ROS levels were measured after treatment of 2-DG or API-2 using DCDHF-DA fluorescent dye, and analyzed by flow cytometry. Values are expressed as means ± SD and two independent experiments were done in triplicate. *, *P<0.05*. ***f***, Δψm was determined after staining cells with a membrane potential sensitive dye, JC-1 and analyzed by Flow cytometry. Box, The ratio of red (J-aggregates) and green (JC-1 monomer) fluorescence intensity. Values are expressed as means ± SD, and experiments were done in triplicate. *, *P<0.05*. ***g***, β-catenin signal pathway in ESCC. NEFH loss after promoter methylation activates Akt that inhibits Gsk3β. Subsequently, increased β-catenin induces β-catenin/TCF-depenendent transcription [29], leading to transcription of specific genes which are involved in mitochondrial function and glycolysis, such as PDH and PK-M2.

Activation of PI3K/Akt and NF-*κ*b increases the overall rate of glycolysis and cell survival [54], [61]. To examine whether the inhibition of these pathways could suppress cell growth, cells were treated with LY294002 (10 µM), a reversible PI3K inhibitor, API-2 (10 µM), an Akt inhibitor, and QNZ (50 µM), a NF-*κ*b inhibitor, for 20 hrs. As shown in Figure S6 *b*, N20 cells were more sensitive to these inhibitors than C2 cells. Among these inhibitors, API-2 was the one that could block Akt activation in both C2 and N20 cells (Fig. 6*a*). QNZ even increased Akt phosphorylation in C2 cells and more dramatically in N20 cells. LY294002 and Wortmannin (1 µM), an irreversible PI3K inhibitor, had little influence on phospho-Akt levels in N20 cells (Fig. S6 *f*). In C2 cells, the Akt level was increased by all of these inhibitors but little difference was observed in PK-M2 and PDH levels compared to untreated control. In N20 cells, the levels of Akt, β-catenin and PK-M2 were decreased, but the expression of Gsk3β and PDH were increased by the treatment of these inhibitors. Interestingly, decrease of Gsk3β phosphorylation and increase of β-catenin phosphorylation at Ser33/Ser37/Thr41 and at Thr41/Ser45 were caused only by API-2. In addition, β-catenin phosphorylation at Ser675 and PK-M2 expression were most decreased by API-2. Therefore, API-2 was most effective at inhibiting Akt-β-catenin signaling among the three inhibitors tested. Moreover, a decrease of Gsk3β phosphorylation and an increase of PDH by 2-DG treatment occurred specifically in N20 cells. The complete abolishment of PK-M2 by treatment with 2-DG was observed only in N20 cells, and thus, 2-DG and API-2 were selected for further study.

Treatment with 2-DG or API-2 in N20 cells markedly decreased anchorage-independent cell growth (Fig. 6*c*). The lactate concentration after 8 hrs of treatment significantly decreased (Fig. 6*d*), whereas ROS levels increased in N20 cells (Fig. 6*e* and Fig S6*d*). The treatment of 2-DG or API-2 in N20 cells had little effect on O_2_ consumption or ATP synthesis (Fig. S6 *c*). Interestingly, alterations in Δψm by 2-DG or API-2 treatment were observed only in N20 cells where ψm significantly increased (Fig. 6*f*, box); in C2 cells, few changes in the distribution of J-aggregates (FL2-H channel) or JC-1 monomers (FL1-H channel) were observed (Fig. 6*f*). In contrast, the distribution of J-aggregates in N20 cells was markedly shifted toward more energized mitochondria by 2-DG or API-2 treatment, whereas that of JC-1 monomers was decreased. These results indicate that cancer cells with loss of NEFH are more susceptible to inhibitors that decrease Akt activation and glycolysis.

## Discussion

Increased expression and nuclear localization of β-catenin protein were reported in ESCC [27], [31]–[33]; however, the mechanism of β-catenin accumulation was unknown in the absence of mutations of either β-catenin or the adenomatous polyposis coli (APC) [27]–[30]. Here, we show that NEFH is a tumor suppressor gene, and that promoter methylation of NEFH gene decreases gene expression and increase the activity of β-catenin. Even though precise mechanisms on how NEFH regulates b-catenin are still needs to be further elucidated, epigenetic silencing of the NEFH gene in ESCC seems to be responsible for increased β-catenin expression, leading to activation of β-catenin/TCF-dependent transcription and key downstream effectors, leading to ESCC tumorigenesis.

The nuclear localization of β-catenin is controlled by its phosphorylation state at NH_2_-terminal serine threonine residues regulated by Gsk3β [62]. Phosphorylation of β-catenin by Gsk3β targets β-catenin to ubiquitination and proteasome degradation. Thus, activation of the pathway represses β-catenin degradation, resulting in nuclear accumulation of β-catenin. In the nucleus, the β-catenin-TCF/Lef complex activates target genes such as c*-Myc* and cyclin D1, which are involved in oncogenic transformation [4], [20]–[26]. Previous studies have shown that TCF-dependent transcriptional activity in ESCC cell lines are increased by mutant β-catenin as compared to wild-type counterparts [24]. Our results clearly show increased β-catenin/TCF-dependent transcriptional activity in NEFH-deficient cells.

Increased aerobic glycolysis is a common abnormality, and alteration of the bioenergetic phenotype of mitochondria is a hallmark of esophageal cancer. When cancer cells generate ATP due in part to mitochondrial respiration injury, they adapt alternative metabolic pathways, such as increasing glycolytic activity, to maintain their energy supply (the Warburg effect). Oncogenic alterations that increase Akt kinase activity stimulate aerobic glycolysis (increased glucose transport and lactate production [54], [59], [63], but reduce the cellular activity of mitochondria in many cancers [64], [65]. However, the molecular pathways underlying alterations in energy metabolism and increased dependency on glycolysis have not been fully delineated. Since malignant cells are highly dependent on the glycolytic pathway for survival and the metabolic alterations make cells resistant to therapeutic agents, understanding and targeting this pathway is an important therapeutic strategy. Several agents including 2-deoxyglucose, have been known to abolish ATP generation through the glycolytic pathway.

In the present study, we demonstrated that loss of NEFH resulted in a Akt-β-catenin-dependent reprogramming of glucose and energy metabolism that included increased glycolysis accompanied by a reciprocal decrease in cellular respiration. NEFH could suppress PK-M2 and induce PDH when wt-β-catenin was expressed in NEFH-deficient cells whereas NEFH could not inhibit the activity of β-catenin mutated in the Gsk3β-targeting Ser33 region. These results indicate that PDH induction and PKM2 repression by NEFH are mediated through Gsk3β-mediated degradation of β-catenin.

We found that β-catenin was required for the inverse regulation of PK-M2 and PDH by cooperating with deregulated NEFH. Interestingly, we found potential TCF-binding elements (TBE) in the PK-M2 and PDH promoters. Five TBE were located 3-Kb upstream of the TSS in the PDH promoter, and three TBE 5-Kb upstream of the TSS in the PK-M2 promoter. Sequences of these TBE matched the consensus for TCF-binding CTTTG(A/T)(A/T) or the inverted sequences (A/T)(A/T)CAAAG [66], [67]; CTTTGAT (−639 bp), CTTTGAA (−1342 bp), CTTTGAA (−1355 bp), CTTTGTA (−2148 bp) and an inverted match TTCAAAG (−2921 bp) in the PDH promoter, and three inverted matches TACAAAG (−3374 bp), TTCAAAG (−3859 bp), and AACAAAG (−4836 bp) in the PK-M2 promoter. These results suggest that PDH and PK-M2 may be targets of β-catenin-TCF/Lef signaling. Future work will elucidate whether the sequences and position of these TBE determine responsiveness to β-catenin/TCF for PDH and PK-M2.

NEFH deficiency increases cellular resistance to oxidative stress (H_2_O_2_) and the mitochondrial membrane potential disrupting agent (Valinomycin) (Fig. S4 *a*–S4*c*), but increases cellular sensitivity to 2-DG, a glycolysis inhibitor. Akt/protein kinase B signaling inhibitor-2 (API-2, Triciribine), which is currently in clinical trials [68] also inhibits glycolysis without affecting cellular respiration. Previous results showed that API-2 inhibits Akt2 activation and harbors anti-tumor activity against tumors with activated Akt [68]. Consistently, we observed increased expression of Akt2 in a clone of NEFH-deficient ESCC cells (N20), and inhibition of anchorage-independent cell growth by API-2. Akt was activated by 2-DG treatment in both C2 and N20 cells (data not shown), but it is unrelated to glycolysis inhibition of 2-DG [69]. Therefore, a combination of API-2 in 2-DG-based therapy might further improve the therapeutic efficacy of 2-DG-mediated growth inhibition.

In addition, mitochondrial metabolism and biogenesis are actively repressed in ESCC cells with loss of NEFH. NEFH loss activates Akt, and further inhibits the activity of Gsk3β, leading to expression of β-catenin in ESCC. Subsequently, Akt/β-catenin signaling positively regulates PK-M2 expression and negatively regulates PDH expression, which might further promote the progression of ESCC. These findings indicate the involvement of the Akt/β-catenin pathway directly in mitochondrial function and identify a new regulatory network that inhibits mitochondrial respiration. Moreover, other major cancer types display NEFH promoter methylation (data not shown). Specific targeting of cellular survival and stress resistance due to NEFH deficiency represents a new rationale therapeutic strategy in human cancer.

## Methods

### Cell Lines and Tissues

Twelve ESCC cell lines, TE1, TE2, TE3, TE4, TE5, KYSE30, KYSE70, KYSE140, KYSE150, KYSE200, KYSE410, and KYSE520 were obtained from the Cell Response Center for Biomedical Research Institute, Department of Aging and Cancer, Tohoku University (TE series) and kindly provided by Dr. Shimada in the Department of Surgery, Kyoto University (KYSE series). Cells were grown in RPMI 1640 medium (2 mg/ml D-glucose) supplemented with 10% fetal bovine serum. HEK293 were obtained from ATCC and grown in DMEM supplemented with 10% FBS. SH-SY5Y human neuroblastoma cells were grown in Phenol red-free RPMI1640 medium containing 10% FBS and 1% MEM non-essential amino acids. Twenty paired ESCC and normal esophagus (patient No. 1–20) tissues were obtained from the Gastroenterology Division, Department of Medicine, University of Maryland. Forty-nine cases of primary ESCC genomic DNA and six ESCC tissue cDNA (patient No. 81–86) were obtained from patients who underwent surgery at the Medical Institute of Bioregulation Hospital, Kyushu University and the Saitama Cancer Center. Fifteen normal esophageal epithelial tissues (NN) were obtained from patients without cancer at Department of Pathology, The Johns Hopkins University.

Written informed consent was obtained from the patients who provided tissue DNA, and this study was approved by the Institutional Review Board of the Johns Hopkins University.

Bisulfite Treatment, Sequencing, Conventional methylation-specific PCR and Quantitative methylation-specific PCR (TaqMan-MSP) are described in the Text S1 with the criteria to determine methylation in cell lines and tissues. Primer sequences are shown in Table S4.

5-Aza-dC/TSA treatment, RT-PCR and Quantitative RT-PCR are described in the Text S1.

### Immunohistochemistry

Three ESCC tissue arrays; esophagus cancer test tissue array with self-matching normal adjacent tissue (ES241), esophagus cancer with matched normal adjacent tissue array (ES801) and esophagus cancer progression tissue array containing normal, inflammation, hyperplasia, squamous and adenocarcinoma (ES804) were purchased from Biomax Inc. (Rockville, MD). After deparaffinization and re-hydration, tissue slides were incubated with anti-NEFH rabbit polyclonal antibody (1∶150 dilutions, Sigma-Aldrich) at 4°C overnight. Secondary antibody was used at a dilution of 1∶500 and incubated for 1 hr. After washing, tissues were stained with freshly prepared DAB solution (DAKO, Carpinteria, CA). Control tissue was incubated with a rabbit IgG control. Sections were counterstained in Mayer's Hematoxyline (Sigma-Aldrich).

### Establishment of shRNA Stable Cell Lines

Stable transfectants expressing siRNA targeting NEFH and non-targeting control were constructed in KYSE30 cells. The GIPZ lentiviral shRNAmir plasmid targeting NEFH and non-silencing verified negative control plasmid (Openbiosystems, Huntsville, AL) were transfected using Arrest-In™ transfection reagent according to the manufacturer's protocol. Puromycin-resistant control or NEFH shRNA clones were selected according to tGFP expression under fluorescence microscope (Nikon TE200, HG-100W mercury lamp) for establishing stable cell lines. Finally, a control (C2) and two NEFH shRNA clones (N12 and N20) were selected based on the expression of NEFH which was confirmed by RT-PCR, real time-RT-PCR, green fluorescence imaging and western blotting.

### Mouse Xenograft Assay

Athymic nude mice were divided into two groups (n = 6 for the C2 *vs.* N12 group, and n = 11 for the C2 *vs.* N20 group) and injected subcutaneously on the left (C2 cells) and right (N12 or N20 cells) flanks with 5 X 10^6^ cells/200 µl PBS per flank by using a 1-ml syringe fitted with a 27-gauge needle. Tumor size was documented by direct measurement in two directions by using Pro-Max calipers (Fowler Instruments, Newton, MA), and the measurements were recorded as tumor volumes (V, mm^3^). Tumor volume was calculated by the equation of *V  =  (L X S^2^)/2*, (L, long diameter, S, short diameter), and was measured twice a week. Statistical significance was determined with a *T-*test. All animals were maintained in accordance with the guidelines of the Johns Hopkins University Animal Care and Use Committee and the National Research Council.

### Construction of NEFH Expression Plasmid and Transfection

The NEFH-pCMV-SPORT6 clone was purchased from ATCC (Manassas, VA), and digested serially with *EcoR1* and *Xba1*. The insert was subcloned into pcDNA3.1 after digestion of pcDNA3.1 with the same enzymes. Cells were transfected using Fugene-6 (Roche, Basel, Switzerland) in OPTI-MEM (Invitrogen) or using Amaxa nucleofector II system (Amaxa Inc. Gaithersburg, MD) per the manufacturer's instructions. The expression of NEFH in the cell line was confirmed by RT-PCR, real time RT-PCR, and Western blot analysis 48 or 72 hrs after transfection. Cells were plated on 24-well plates at a density of 3 X 10^4^ cells/well and incubated overnight at 37°C. Next day, cells were transfected with 0.5 µg of pNEFH or mock vector. After incubation for 72 hrs, the tetrazolium-based cell viability (MTT) assay was performed.

### Mitochondrial Membrane Potential (Δψm)

Fluorescence imaging was performed by staining mitochondria with the membrane potential sensitive dye JC-1 (Invitrogen). After incubation with JC-1 (30 min at room temperature), mitochondria were washed to remove excess dye by a gentle centrifugation step (3 min at 2000 x g). For measuring the mitochondrial membrane potential (Δψm), fluorescence was acquired by flow cytometry: green fluorescence at FL1-H channel and red fluorescence at FL2-H channel. The ratio of red to green fluorescence was calculated for Δψm. As an alternative, cells (5 × 10^4^ cells) in attachment in 96-well culture plate were assayed using a fluorescence microplate reader (Molecular Devices) and fluorescence was acquired at 490/530 (ex/em) for JC-1 monomers and 525/590 (ex/em) for J-aggregates. Δψm was calculated using the ratio of fluorescence of J-aggregates to JC-1 monomers.

### Total Cellular Oxygen Consumption and ATP Production

Equal numbers of cells (6 × 10^6^) were suspended in 600 µl of growth medium and transferred into the chamber of an Oxytherm electrode unit (Hansatech Instrument Ltd.), which uses a Clark-type electrode to monitor the dissolved oxygen concentration in the sealed chamber over time. The data were exported to a computerized chart recorder (Oxygraph, Hansatech Instrument Ltd.), which calculates the rate of O_2_ consumption (nmole/min/10^7^). The temperature was maintained at 37°C during the measurement. Intracellular ATP levels were measured for an equal number of cells (2 ×10^5^) using an ATP assay kit (Sigma) according to the manufacturer's instructions. After addition of the assay mixture containing luciferin and luciferase, luminescence was measured immediately using a Wallace microplate luminescence reader (Perkin Elmer, Waltham, MA). Results are presented as arbitrary fold.

### Reactive Oxygen Species

The level of ROS in cells was measured using 5-(and-6)-chloromethyl-2′,7′-dichlorodihydrofluorescein diacetate, acetyl ester (DCDHF-DA) (Invitrogen). In brief, cells were incubated with 2.5 µM DCDHF-DA for 30 min at 37°C. After trypsinization, single cell suspensions were subjected to flow cytometry. As an alternative, cells in culture plate were assayed using a fluorescence microplate reader and fluorescence was acquired at 495/525 (ex/em).

### Glucose Consumption and Lactate Production

Cells were seeded at a density of 1 × 10^5^/well and incubated overnight. Culture medium was changed with fresh, growth medium before further incubation for 16 hrs. The culture medium was then collected, and filtered through a 0.22 µm pore membrane. Glucose and lactate levels were measured using glucose and lactate assay kits as manufacturer's instructions (Biovision, Exton, PA). Glucose consumption was determined from the difference in glucose concentration compared with control. A MTT assay was performed to verify equal number of cells, and no significant difference was found (data not shown).

### Luciferase Reporter Assay

β-catenin/Tcf transcriptional reporter activity was performed as described by using TOPflash/FOPflash TCF Reporter Kit (Upstate Biotechnology, Lake Placid, NY). TOPflash has four copies of wild-type Tcf binding site, and FOPflash has three copies of mutant TCF binding site upstream of the E1B TATA box and Luciferase open reading frame. FOPflash was used as a control for measuring nonspecific activation of the reporter. Cells were transfected with reporter plasmid (0.1 µg) and a *Renilla* luciferase (pSV-Renilla, 0.02 µg (Promega) (an internal control for transfection efficiency) using FuGene HD reagent (Roche, Indianapolis, IN). After 48 hrs of incubation, the reporter activity was measured using the Dual-luciferase reporter assay system (Promega). Relative luciferase activity (arbitrary units) was reported as fold induction after normalization for transfection efficiency. Transfections were performed in triplicate and repeated twice to ensure reproducibility.

### Western Blot Analyses

Whole cell lysates extracted in RIPA buffer were separated on 4–12% gradient SDS-PAGE and transferred to nitrocellulose membrane. The blots were incubated with specific antibodies for each gene for 2 h at room temperature or 4°C overnight. After antibody washing, the blots were reacted with their respective secondary antibody and detected with enhanced chemiluminescence reagents (Amersham, Pittsburgh, PA) according to the supplier's protocol. Antibodies specific for NEFH recognizing both dephosphorylated and phosphorylated form of NEFH and for β-actin were purchased from Sigma. All antibodies were purchased from Cell Signaling Technologies (Beverly, MA) except for the anti-Hsp60, anti- mitochondrial H^+^-ATPase, anti-nuclear protein 98, and anti- PDK1 antibodies (Stressgen, British Colombis, Canada). Anti-PK-M2 antibody was from Abnova (Walnut, CA), anti-bcl-2 antibody from Santa Cruz Biotechnologies (Santa Cruz, CA), and anti-Bax antibody from Neomarkers (Fremont, CA).

### Knockdown of PTEN, Gsk3β and β-Catenin

A siRNA pool targeting PTEN, Gsk3β and β-catenin were purchased from Dharmacon, and transfected into C2 and/or N20 cells as described. Cells were incubated for 48 hrs after transfection, and total cellular oxygen consumption, ATP production, Δψm, ROS level, lactate production were examined. For the lactate assay, cells were incubated for 24 hrs and cell culture medium was collected. pCIneo-β-cat expressing wild-type β-catenin and pCIneo-β-cat-S33Y expressing mutant β-catenin that has a defect in Gsk3β-dependent phosphorylation of β-catenin was kindly given by Dr. Bert Vogelstein from the Johns Hopkins University.

### Statistical Analysis

We used the methylation levels (TaqMeth V) for NEFH to construct Receiver Operating Characteristic (ROC) curves for the detection of ESCC. In the ROC analysis, a tangent point where the slope of the ROC curve was 1.00 has been selected as an optimal cut-off point to balance sensitivity and specificity. P value was derived from Z value that was calculated from the equation of (AUROC-0.5)/Std Err (standard error of AUROC). Using this approach, the AUROC (Area under ROC) identified optimal sensitivity and specificity levels (i.e., cut-offs) to distinguish normal from malignant ESCC tissues. The cut-off value (0.985) determined from the ROC curve was then applied to determine the frequency of gene methylation. Samples with a methylation level higher than the cut-off were designated as methylated, and samples with a methylation level lower than the cut-off were designated as un-methylated. The significance level used was 0.05 and all statistical analyses were conducted using STATA Version 9 (STATA Inc., College Station, TX).

## Supporting Information

Text S1 (0.07 MB DOC)

Figure S1 Analysis of NEFH methylation in ESCC. a, One dense CpG island (colored area) resides 300 bp upstream of the TSS in the promoter region of NEFH and another 1.1 Kb downstream of the TSS. Primers for bisulfite-sequencing (Bi-F1 and Bi-R1), MSP and TaqMan-MSP were designed within the region which covered most of the CpG-rich region proximal to the TSS ({similar, tilde operator } 400 bp) in the NEFH promoter. F, forward; R, reverse. TSS, transcription start site. P, the probe for TaqMan-MSP. A total of 36 CGs were numbered from the first to last CG in the sequences as indicated. Individual CpG methylation in cell lines (b) and primary ESCC (PT) with their corresponding normal esophageal tissues (PN) is shown (c). HEK293 human embryonic kidney cell line was included to compare CpG methylation between cancer and non-tumorigenic cell lines. CpGs undetermined were not squared. Black square, methylated CpG; white square, unmethylated CpG; shaded square, partially methylated CpG. The criteria to determine methylation in individual CpG are described in the Supplemental Methods. When analyzed in the region downstream of the TSS (indicated as Bi-F3 and Bi-R2) by bisulfite-sequencing, NEFH methylation was observed in normal tissue samples collected from ESCC patients and HEK293 cells as well as 12 ESCC cell lines, indicating that NEFH methylation in the promoter region upstream of the TSS discriminates normal and tumor tissues. d, Bisulfite-sequencing results of the NEFH promoter in 12 ESCC cell lines, HEK293 cells, and primary ESCC (PT) together with their corresponding normal esophageal tissues (PN). Black square, methylation; white square, no methylation. e, Representative results of NEFH bisulfite-sequencing in cell lines and tissues. All guanines present after sequencing that are complementary to methyl cytosines on the opposite DNA strand. Arrow, methylated CpGs maintained after bisulfite treatment. f, Promoter methylation of NEFH in ESCC cell lines was further confirmed by combined bisulfite restriction analysis (COBRA) after gel-extraction of the PCR product of bisulfite-treated DNA. Only the PCR products of the methylated alleles are cleaved by the enzyme BstUI that recognizes the sequence CGCG, not CUCU. Samples were loaded on a 10% acrylamide gel, stained with 1X SYBR Green Gold and visualized under UV light. L, 1 Kb Plus DNA ladder. Arrows, 100 bp.
(16.14 MB TIF)

Figure S2 Establishment of NEFH or control shRNA stable clones. Two stable clones expressing low levels of NEFH (N12 and N20) and a non-targeting control clone (C2) were established in KYSE30 cells for further study by selection of GFP-expressing, puromycin-resistant cells after transfection of shRNA plasmid to inhibit the endogenous NEFH expression (Material and Methods). NEFH-knockdown at the mRNA level was confirmed by RT-PCR (a) and real-time RT-PCR analysis (b), and at the protein level by fluorescence microscopy and by western blot analysis (c). The knockdown of NEFH was greater in the N20 than in the N12 clone. d, To confirm NEFH expression, IHC analysis was performed in tissue sections of tumor xenografts dissected from mice. Expressions of β-catenin, PK-M2 and PDH in tumor xenografts were consistent with those observed in protein lysates from cell culture as shown in Figure 3. Scale bar, 10 µm.
(3.60 MB TIF)

Figure S3 Activation of β-catenin-TCF/Lef signaling by NEFH-knockdown. a, The slight increase of total Akt in NEFH-deficient cells was due to increased Akt1 and Akt2 in N12 and N20 cells, respectively. b, Basal expression of phospho-Akt and β-catenin was examined in ESCC cell lines. Cell lysates from ESCC cell lines were run in 4-12% polyacrylamide gel and transferred onto nitrocellulose membrane. Cell lysate from SH-SY5Y was loaded together to compare NEFH level with those in ESCC cell lines. Exposure time of the protein membrane on X-ray film after extensively washing was 10 sec (short) and 1 min (long). Faint expression of NEFH was detected in TE series by relatively long exposure (1 min) of the protein membrane reacted with a specific anti-NEFH antibody. No mutation of exon 3 of the β-catenin was observed in all 12 ESCC cell lines (data not shown). c, NEFH expression was undetectable in KYSE140 cells that harbored NEFH promoter methylation (Fig. 1). The NEFH promoter was not methylated in HEK293 cells and SH-SY5Y a neuroblastoma cell line (determined by Bisulfite-sequencing analysis), and high levels of NEFH were detected in these cell lines. KYSE140 cells were transfected with pcDNA3.1 (mock) or NEFH expressing plasmid (pNEFH). Interestingly, phospho-Akt and β-catenin levels seemed to be inversely correlated with NEFH expression. Gsk3β expression was positively correlated with NEFH level in KYSE30 and KYSE140 cells. d, HEK293 cells were transfected with NEFH-siRNA and non-targeting control, and total cell lysates were extracted for Western blot analysis. β-actin is a loading control. Increased cell proliferation was observed in HEK293 cells transfected with siRNA targeting NEFH (data not shown). Real-time RT-PCR was performed using cDNA prepared from C2, N12 and N20 (e) or KYSE140 cells (f) 72 hrs after transfection using TaqMan pre-designed primers and probes as described in Methods. Transcriptional level of each gene was normalized by the level of β-actin. NEFH deficiency increased mRNA expression of β-catenin and downstream target genes (TCF1, cyclin D1, and MMP-7). Values are expressed as means ± SD, and experiments were done in triplicate, and repeated twice. Expression of NEFH was confirmed by RT-PCR (Box).
(1.67 MB TIF)

Figure S4 Cellular resistance to oxidative stress by NEFH-knockdown. a, Cellular resistance to oxidative stress was increased by down-regulation of NEFH. KYSE30 cells (left) and HEK293 cells (right) were transfected with NEFH- (N) or control-siRNA (C) and exposed to H2O2 (0 {similar, tilde operator } 400 µM) in serum-free medium for 2 hrs. Cells were then recovered from oxidative stress by further incubation in growth medium for 24 (KYSE30, left) or 48 hrs (HEK293, right). KYSE30 cells were more sensitive to oxidative stress than HEK293 cells, since the sensitivity of KYSE30 cells to H2O2 treatment was seen in 24 hrs of recovery whereas that of HEK293 cells was observed in 48 hrs of recovery. In the presence of NEFH knockdown, an increased cell survival to H2O2 exposure was seen in both cell types, indicating cellular resistance to oxidative stress by down-regulation of NEFH. Data are expressed as % of untreated control. Values are expressed as means ± SD, and experiments were repeated twice in triplicate. b, KYSE140 cells were transiently transfected with NEFH expressing plasmid or control plasmid (p3.1) and treated with or without H2O2 (100 µM) in serum-free medium for 2 hrs. After 24 hrs of recovery, cell viability was examined. In the absence of H2O2 treatment, the viability in cells expressing NEFH was about 70% of control, whereas in the presence of H2O2 treatment, the decrease of cell viability was further enhanced to 50% of control. These results suggest that NEFH sensitizes cells to oxidative stress. *, P<0.05 (T-test). c, Valinomycin is a potassium ionophore that collapses mitochondrial membrane potential, and loss of mitochondrial membrane potential is observed in the early stages of apoptosis. HEK293 cells transfected with NEFH- (N) or control-siRNA (C) were treated with Valinomycin (0 {similar, tilde operator } 10 nM) for 16 hrs, and cell survival was examined. Down-regulation of NEFH by siRNA transfection increased cell survival against Valinomycin-induced apoptosis, indicating that loss of NEFH in ESCC promotes cellular resistance to oxidative stress and mitochondrial dysfunction. d, Δψm was determined by flow cytometry after staining C2 and N20 cells with a membrane potential sensitive dye, JC-1 (upper). Quadrant statistics for the population of cells that resided at UR and LR (lower). e, The red (J-aggregates) and green (JC-1 mononer) fluorescence intensities were analyzed by flow cytometry, and the ratio of red to green fluorescence was calculated. Values are expressed as means ± SD, and experiments were done in triplicate.
(3.43 MB TIF)

Figure S5 β-catenin knock-down inhibits cell growth. a, Δψm was determined after staining cells with a membrane potential sensitive dye, JC-1. Fluorescence intensity was acquired by reading cells in a fluorescence microplate reader. Values are expressed as means ± SD, and experiments were done in triplicate. b, Cellular growth was evaluated by the MTT cell growth assay for 4 days after transfection of β-catenin siRNA. No significant difference in cell growth was found in 24 hr, but cell growth was inhibited in both C2 and N20 cells by the β-catenin knockdown after 3 days of incubation. Data are expressed as absorbance at 570 nm and experiments were repeated twice in triplicate.
(1.46 MB TIF)

Figure S6 Cells with low expression of NEFH are more sensitive to API-2 or 2-DG. a, Cells were incubated in complete growth medium (2 mg/ml glucose) in the presence of 2-DG (0 {similar, tilde operator } 3 mg/ml) or were incubated under glucose-free condition for 24 hrs, and cell viability was assessed by the MTT assay. Values are expressed as means ± SD of absorbance at 570 nm (left), or % of control (no treatment) in each cell line. Experiments were repeated twice and done in triplicate. *, P<0.05. b, Cell viability was evaluated 24 hrs after the treatment of inhibitors. UC, untreated control; LY, LY294002 (10 µM); API-2 (10 µM); QNZ (50 µM). c, Oxygen consumption (left) and ATP levels (right) were measured after treatment of 2-DG or API-2. d, Representative results of ROS imaging after 2-DG or API-2 treatment in C2 and N20 cells. e, In 8 hrs, cell viability was not significantly decreased by 2-DG or API-2 treatment (left, MTT assay), but inhibition of cell viability was observed at 24 hrs of treatment (right, coulter counting). f, C2 and N20 cells were treated with LY294002 (10 µM) and Wortmannin (1 µM) for 2 and 20 hrs, and western blot analysis was performed. g, C2 cells were pre-treated with LY294002 and Wortmannin for 1 hr, and EGF (10 ng/ml) was added. Cells were then further incubated for 2 hrs, and western blot analysis was performed. Similar results were observed in cells treated with inhibitors for 16 hrs (data not shown).
(10.30 MB TIF)

Table S1 (0.03 MB XLS)

Table S2 (0.05 MB XLS)

Table S3 (0.04 MB XLS)

Table S4 (0.03 MB XLS)
