# Supplementary material for: Neurofilament Heavy Polypeptide Regulates the Akt-β-Catenin Pathway in Human Esophageal Squamous Cell Carcinoma
Source: PLoS One. 2010 Feb 3;5(2):e9003. doi: 10.1371/journal.pone.0009003 (PMC2815775; doi:10.1371/journal.pone.0009003)
Supplement: Text S1 — (0.07 MB DOC) [file pone.0009003.s001.doc]

**Supplemental Methods**

***Inhibitors*.** API-2 (Triciribine), an inhibitor of cellular phosphorylation/activation of Akt1/Akt2/Akt3 by targeting an Akt effector molecule, was purchased from Calbiochem (San Diego, CA). 2-Deoxyglucose (2-DG), LY294002 (PI3K inhibitor), Wortmannin (PI3K inhibitor), and QNZ (6-Amino-4-(4-phenoxyphenylethylamino) quinazoline, NF*κ*-b inhibitor) were purchased from Sigma-Aldrich (Saint Louis, MO).

***Bisulfite Treatment and Sequencing*.** Bisulfite treatment of genomic DNA, PCR amplification, and sequencing were performed as described [71].

***The criteria to determine methylation in cell lines and tissues*.** Bisulfite-sequencing was based on nucleotide sequences in electropherograms. When only a cytosine or a thymidine peak existed in a CpG, the sequence was “CG” (100% methylation) or “TG” (0% methylation). When both methylated and unmethylated alleles were observed in a CpG sequence, it was considered as “partially methylated” (M/U). When a “partial methylated CpG” was observed, a cytosine peak was compared to a thymidine peak in the CpG. If a cytosine peak was similar to a thymidine peak or dominant, the sequence in electropherograms was “NG” or “CG”, indicating that over 50% methylated alleles existed. When a thymidine peak was dominant, the sequence was “TG”, indicating less than 50% methylated alleles. Only “NG” and “CG” were considered as “methylated” in the CpG. When “methylated” CpG was found in more than 25% of total CpGs in an amplified PCR product, it was considered as “methylation-positive.” A search for CpG islands in each gene promoter was done by using the on-line accessible software, Methprimer.

***Methylation-specific PCR*.** Bisulfite-treated DNA was amplified with either methylation-specific or unmethylation-specific primer sets for NEFH. PCR reactions were performed for 35 cycles of 95 °C for 30 sec, 60 °C for 30 sec, and 72 °C for 30 sec.

***Quantitative methylation-specific PCR (TaqMan-MSP*).** For quantitative methylation analysis, TaqMan-MSP was performed as reported [71], and all reactions were performed in duplicate. The methylation ratio was defined as the quantity of fluorescence intensity derived from the NEFH promoter amplification divided by fluorescence intensity from β-actin amplification, and multiplied by 100 (TaqMan methylation value: TaqMeth V). This ratio was used as a measure for the relative level of methylated DNA in samples. Those samples were categorized as unmethylated or methylated based on optimal cut-offs from ROC analysis.

***5-Aza-dC/TSA treatment and RT-PCR***. Cells were treated with 1 μM or 5  μM 5-aza-2’-deocycytidine (5-Aza-dC) (Sigma-Aldrich) every 24 hrs for 3 days, and then, 300 nM trichostatin A (TSA) (Sigma) was added to the media for the final 24 hrs. RNA extraction and cDNA synthesis were performed as described [71]. For amplification of NEFH, touch-down PCR or standard PCR was performed as reported previously [14]. PCR products were gel-extracted and sequenced to verify true expression of the gene.

***Quantitative RT-PCR*.** cDNAs from an ESCC patient and from a patient without cancer (NN) were purchased from BioChain Institute, Inc. (Hayward, CA). A pair of normal and tumor cDNA was obtained from patients who underwent surgery at the Medical Institute of Bioregulation Hospital, Kyushu University. One μl of each cDNA was used for real-time RT-PCR using QuantiFast SYBR Green PCR Kit (Promega, Valencia, CA) as described previously [71]. Expression of NEFH mRNA relative to control gene (18S rRNA) was calculated based on the threshold cycle (C_t_) as 2-
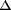
(
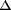
Ct), where
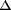
C_t_ = C_t,NEFH_ - C_t,Control_ and
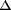
(
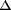
C_t_) =
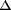
C_t,N_ -
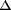
C_t,T_ (N, normal tissue cDNA, T, tumor tissue cDNA). For Quantitative RT-PCR in cell lines, cDNAs from C2, N12, and N20 cells were used for real-time RT-PCR using 2X Master Mix and TaqMan pre-designed primers and probes purchased from Applied Biosystems (Foster City, CA). mRNA expression of each gene relative to control genes (β-actin) was calculated.

***Immunohistochemistry in tumor xenografts*.** Tumor mass was dissected from each mouse flank and processed by formalin fixation, and embedded in paraffin for tissue section on slides. Anti-PCNA antibody was purchased from Cell Signaling (Beverly, MA), and anti-PK-M2 antibody against dimeric M2-PK (Clone DF4) was purchased from ScheBo Biotech (UK Ltd, Basingstoke, UK).

***MTT assay*.** Cells were plated on a 24-well plate at a density of 2 X 10^4^ cells/well and incubated overnight at 37°C. Cells were incubated for 5 days, and the tetrazolium-based cell viability (MTT) assay was performed every day. The results were expressed as absorbance at 570 nm or a percentage of MTT reduction in samples compared to control (100%).

***Chemoinvasion assay*.** Cells were seeded at a density of 5 x 10^3^/well in transwell chamber (Lowell, MA) previously coated with Matrigel (upper part) and type I collagen (lower part). Cells were incubated for 16 hrs. As a chemoattractant, 10% serum or hepatocyte growth factor (HGF, 100 ng/ml) in 1% serum was used for the assay. A total of 10 sites per membrane were randomly selected for cell counting. Simultaneously, equal number of cells was seeded on 24-well plates, incubated for 16 hrs, and MTT assay was performed (data not shown).

***Cell cycle analysis*.** Cells (1 x 10^6^ cells/ml) were starved in serum-free medium for 24 hrs, and were then released in 10% serum-medium for 24 hrs for cell cycle transition. Cells were then fixed in 70% ethanol at 4°C for 1 hr, resuspended in 1 ml PBS containing 20 μg/ml propidium iodide (Sigma-Aldrich, Saint Louis, MO), 20 µg/ml RNase A (Sigma-Aldrich, Saint Louis, MO), incubated for 30 min at room temperature, and analyzed by flow cytometry (FACSCaliber, BD Biosciences, San Jose, CA) for DNA synthesis and cell cycle status. Ten thousand events per sample were collected and analyzed using the Cell-Quest (BD Biosciences).

***Soft agar assay***. Cells (1 X 10^4^) were seeded in 1 ml of 0.3% low-melting agarose over a 0.6% agar bottom layer in McCoy’s, 5X supplemented with 10% FBS and puromycin (0.5 μg/ml). The medium was changed three times a week and the clones were allowed to grow for two weeks. Two independent experiments were performed and each experiment was done in triplicate.

***Colony focus assay***. Colony focus assays were performed in KYSE140 cells after transfection with NEFH expressing plasmid (pNEFH) or pcDNA3.1 (p3.1). Cells were incubated in the presence of G418 (125 μg/ml) for two weeks.

***Trypan blue exclusion assay***. Three days after transfection with pNEFH or p3.1, KYSE140 cells were stained with Trypan blue solution (Invitrogen) for 10 minutes, and viable cells (clear) and apoptotic cells (blue) were counted under microscope.

***Anexin V analysis*.** Apoptosis was quantified using the Anexin V-FITC apoptosis kit (BD Biosciences, San Diego, CA) in accordance with the manufacturer's instructions. Briefly, cells were trypsinized, pelleted by centrifugation, and resuspended in Anexin V–binding buffer (150 mmol/L NaCl, 18 mmol/L CaCl_2_, 10 nmol/L HEPES, 5 mmol/L KCl, 1 mmol/L MgCl_2_). FITC-conjugated Anexin V and 7-amino-actinomycin D dye (7-AAD) were added to cells and incubated for 30 min at room temperature in the dark. Labeled cells were detected by flow cytometry and analyzed with CellQuest software (Becton Dickinson, Mountain View, CA).

***Caspase 3/7 assay***. Cells were cultured in 96-well plates and transfected with pNEFH or p3.1. Then, cells were incubated for 72 hrs and analyzed using Caspase-Glo 3/7 Assay kit (Promega) according to the manufacturer’s instructions.

***Mutational Analysis of β-catenin*.** PCR for exon 3 of β-catenin was performed with gDNA extracted from 12 ESCC cell lines and 69 ESCC tissues. PCR fragments were sequenced with either forward or reverse primer [17].

***Cytoplasmic and nuclear Protein Extraction***. Nonidet P-40 soluble fraction (cytosol) was extracted from cell pellets in buffer A (10 mM HEPES/potassium hydroxide, 1 mM dithiothreitol, 0.1 mM EGTA, 0.1 mM EDTA, 0.5 mM phenylmethylsulfonyl fluoride, and 0.2 mM phenylmethylsulfonyl fluoride, pH 7.9) with 0.5% Nonidet P-40 for 30 min, followed by centrifugation at 1,000 x *g* for 5 min to pellet the nuclei. After separation of the cytoplasmic fraction, Nonidet P-40 soluble fraction (nuclei) was harvested by resuspension in ice-cold buffer B (20 mM HEPES/potassium hydroxide buffer, 0.4 mM sodium chloride, 1 mM dithiothreitol, and 0.2 mM phenylmethylsulfonyl fluoride, pH 7.9). Tubes were incubated for 30 min on ice, vortexed for 1 min, and then centrifuged to clear the cellular debris**. Cytoplasmic** and nuclear extracts were stored at -80°C until use.
