# Supplementary figures and images for: Neurofilament Heavy Polypeptide Regulates the Akt-β-Catenin Pathway in Human Esophageal Squamous Cell Carcinoma
Source: PLoS One. 2010 Feb 3;5(2):e9003. doi: 10.1371/journal.pone.0009003 (PMC2815775; doi:10.1371/journal.pone.0009003)

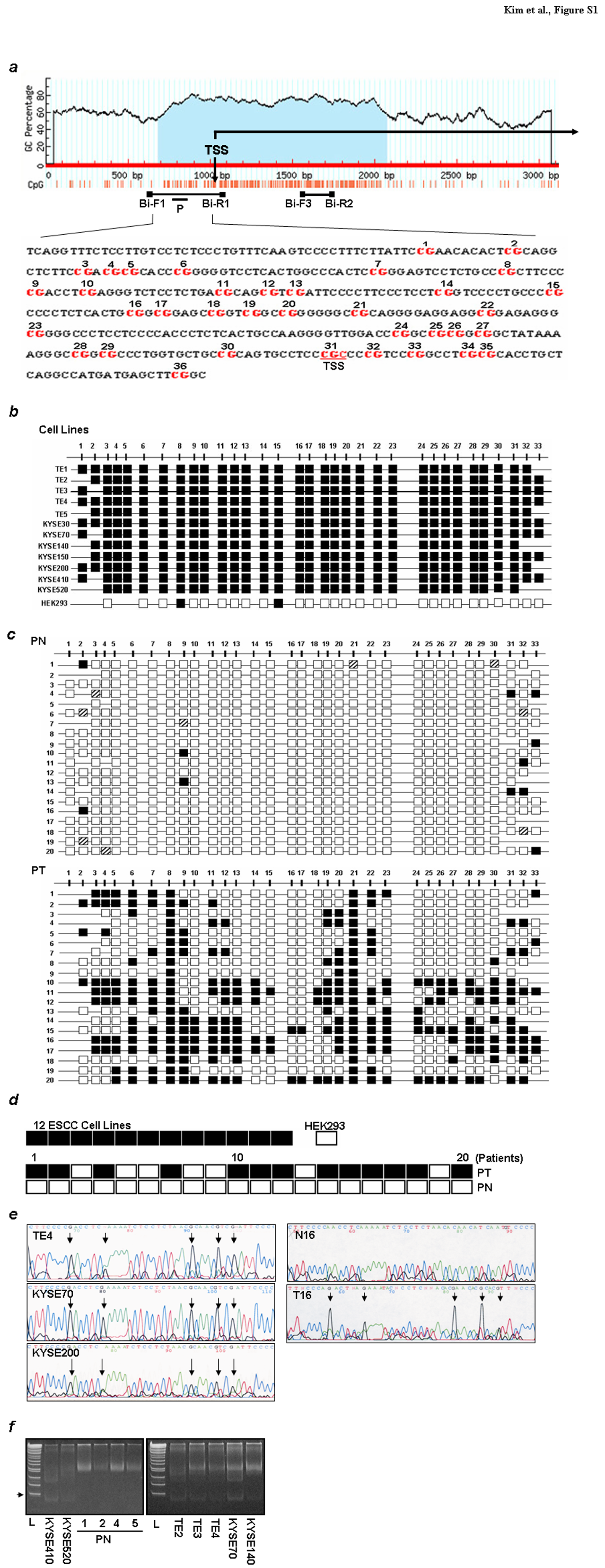

Supplement: Figure S1 — Analysis of NEFH methylation in ESCC. a, One dense CpG island (colored area) resides 300 bp upstream of the TSS in the promoter region of NEFH and another 1.1 Kb downstream of the TSS. Primers for bisulfite-sequencing (Bi-F1 and Bi-R1), MSP and TaqMan-MSP were designed within the region which covered most of the CpG-rich region proximal to the TSS ({similar, tilde operator } 400 bp) in the NEFH promoter. F, forward; R, reverse. TSS, transcription start site. P, the probe for TaqMan-MSP. A total of 36 CGs were numbered from the first to last CG in the sequences as indicated. Individual CpG methylation in cell lines (b) and primary ESCC (PT) with their corresponding normal esophageal tissues (PN) is shown (c). HEK293 human embryonic kidney cell line was included to compare CpG methylation between cancer and non-tumorigenic cell lines. CpGs undetermined were not squared. Black square, methylated CpG; white square, unmethylated CpG; shaded square, partially methylated CpG. The criteria to determine methylation in individual CpG are described in the Supplemental Methods. When analyzed in the region downstream of the TSS (indicated as Bi-F3 and Bi-R2) by bisulfite-sequencing, NEFH methylation was observed in normal tissue samples collected from ESCC patients and HEK293 cells as well as 12 ESCC cell lines, indicating that NEFH methylation in the promoter region upstream of the TSS discriminates normal and tumor tissues. d, Bisulfite-sequencing results of the NEFH promoter in 12 ESCC cell lines, HEK293 cells, and primary ESCC (PT) together with their corresponding normal esophageal tissues (PN). Black square, methylation; white square, no methylation. e, Representative results of NEFH bisulfite-sequencing in cell lines and tissues. All guanines present after sequencing that are complementary to methyl cytosines on the opposite DNA strand. Arrow, methylated CpGs maintained after bisulfite treatment. f, Promoter methylation of NEFH in ESCC cell lines was further confirmed by [file pone.0009003.s002.tif]

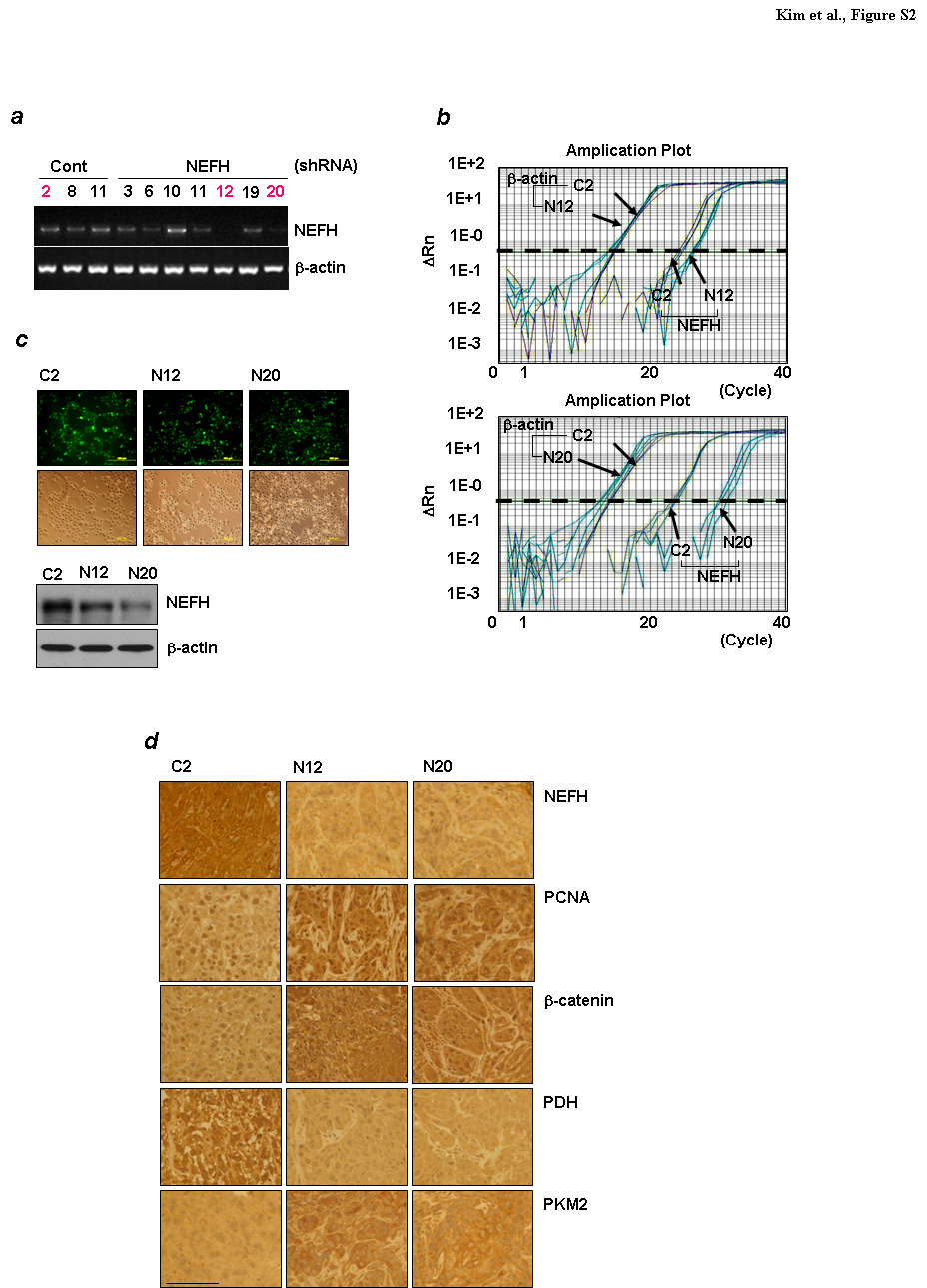

Supplement: Figure S2 — Establishment of NEFH or control shRNA stable clones. Two stable clones expressing low levels of NEFH (N12 and N20) and a non-targeting control clone (C2) were established in KYSE30 cells for further study by selection of GFP-expressing, puromycin-resistant cells after transfection of shRNA plasmid to inhibit the endogenous NEFH expression (Material and Methods). NEFH-knockdown at the mRNA level was confirmed by RT-PCR (a) and real-time RT-PCR analysis (b), and at the protein level by fluorescence microscopy and by western blot analysis (c). The knockdown of NEFH was greater in the N20 than in the N12 clone. d, To confirm NEFH expression, IHC analysis was performed in tissue sections of tumor xenografts dissected from mice. Expressions of β-catenin, PK-M2 and PDH in tumor xenografts were consistent with those observed in protein lysates from cell culture as shown in Figure 3. Scale bar, 10 µm. (3.60 MB TIF) [file pone.0009003.s003.tif]

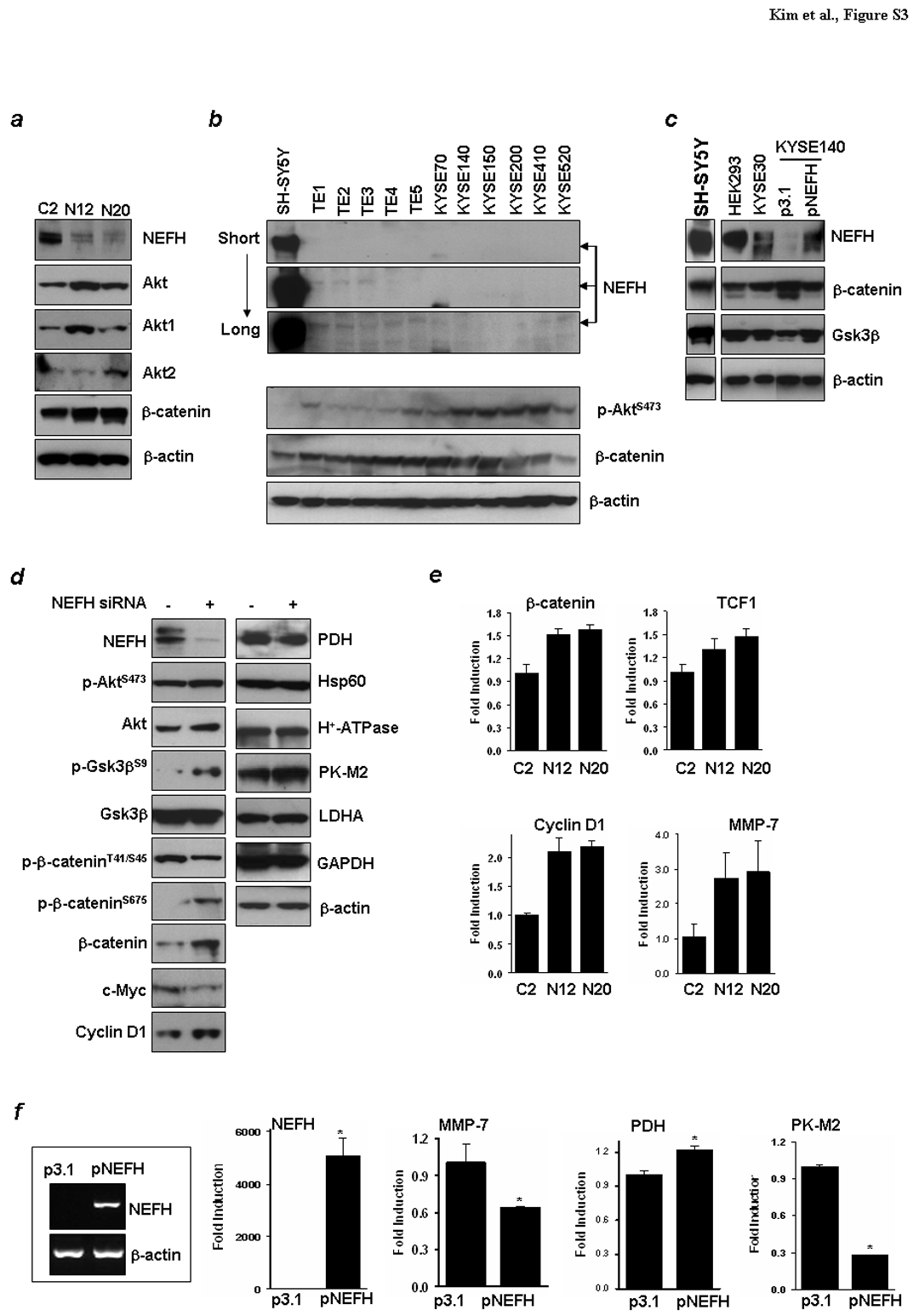

Supplement: Figure S3 — Activation of β-catenin-TCF/Lef signaling by NEFH-knockdown. a, The slight increase of total Akt in NEFH-deficient cells was due to increased Akt1 and Akt2 in N12 and N20 cells, respectively. b, Basal expression of phospho-Akt and β-catenin was examined in ESCC cell lines. Cell lysates from ESCC cell lines were run in 4-12% polyacrylamide gel and transferred onto nitrocellulose membrane. Cell lysate from SH-SY5Y was loaded together to compare NEFH level with those in ESCC cell lines. Exposure time of the protein membrane on X-ray film after extensively washing was 10 sec (short) and 1 min (long). Faint expression of NEFH was detected in TE series by relatively long exposure (1 min) of the protein membrane reacted with a specific anti-NEFH antibody. No mutation of exon 3 of the β-catenin was observed in all 12 ESCC cell lines (data not shown). c, NEFH expression was undetectable in KYSE140 cells that harbored NEFH promoter methylation (Fig. 1). The NEFH promoter was not methylated in HEK293 cells and SH-SY5Y a neuroblastoma cell line (determined by Bisulfite-sequencing analysis), and high levels of NEFH were detected in these cell lines. KYSE140 cells were transfected with pcDNA3.1 (mock) or NEFH expressing plasmid (pNEFH). Interestingly, phospho-Akt and β-catenin levels seemed to be inversely correlated with NEFH expression. Gsk3β expression was positively correlated with NEFH level in KYSE30 and KYSE140 cells. d, HEK293 cells were transfected with NEFH-siRNA and non-targeting control, and total cell lysates were extracted for Western blot analysis. β-actin is a loading control. Increased cell proliferation was observed in HEK293 cells transfected with siRNA targeting NEFH (data not shown). Real-time RT-PCR was performed using cDNA prepared from C2, N12 and N20 (e) or KYSE140 cells (f) 72 hrs after transfection using TaqMan pre-designed primers and probes as described in Methods. Transcriptional level of each gene was normalized by the level of β-actin. NEFH deficie [file pone.0009003.s004.tif]

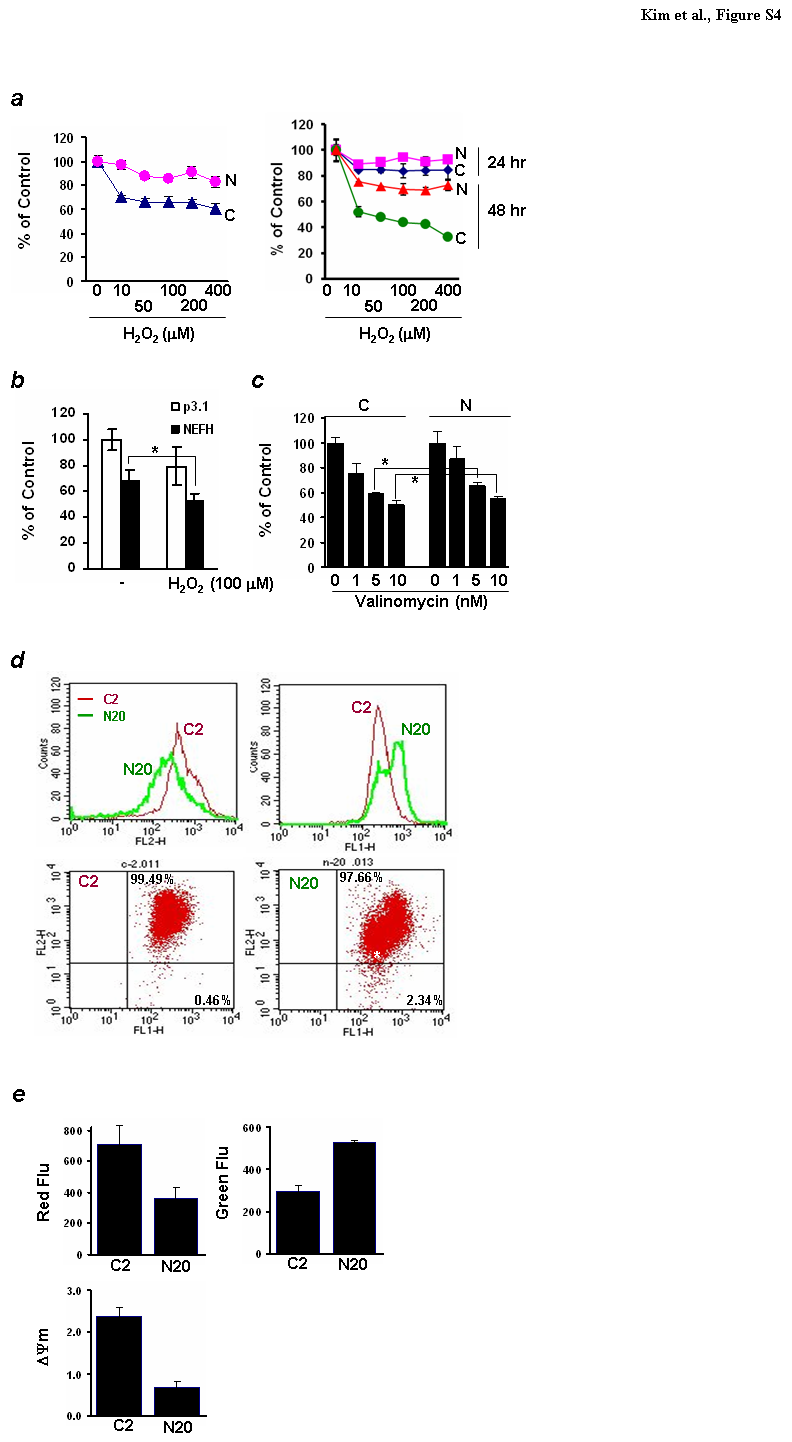

Supplement: Figure S4 — Cellular resistance to oxidative stress by NEFH-knockdown. a, Cellular resistance to oxidative stress was increased by down-regulation of NEFH. KYSE30 cells (left) and HEK293 cells (right) were transfected with NEFH- (N) or control-siRNA (C) and exposed to H2O2 (0 {similar, tilde operator } 400 µM) in serum-free medium for 2 hrs. Cells were then recovered from oxidative stress by further incubation in growth medium for 24 (KYSE30, left) or 48 hrs (HEK293, right). KYSE30 cells were more sensitive to oxidative stress than HEK293 cells, since the sensitivity of KYSE30 cells to H2O2 treatment was seen in 24 hrs of recovery whereas that of HEK293 cells was observed in 48 hrs of recovery. In the presence of NEFH knockdown, an increased cell survival to H2O2 exposure was seen in both cell types, indicating cellular resistance to oxidative stress by down-regulation of NEFH. Data are expressed as % of untreated control. Values are expressed as means ± SD, and experiments were repeated twice in triplicate. b, KYSE140 cells were transiently transfected with NEFH expressing plasmid or control plasmid (p3.1) and treated with or without H2O2 (100 µM) in serum-free medium for 2 hrs. After 24 hrs of recovery, cell viability was examined. In the absence of H2O2 treatment, the viability in cells expressing NEFH was about 70% of control, whereas in the presence of H2O2 treatment, the decrease of cell viability was further enhanced to 50% of control. These results suggest that NEFH sensitizes cells to oxidative stress. *, P<0.05 (T-test). c, Valinomycin is a potassium ionophore that collapses mitochondrial membrane potential, and loss of mitochondrial membrane potential is observed in the early stages of apoptosis. HEK293 cells transfected with NEFH- (N) or control-siRNA (C) were treated with Valinomycin (0 {similar, tilde operator } 10 nM) for 16 hrs, and cell survival was examined. Down-regulation of NEFH by siRNA transfection increased cell survival against Valinomycin-induced apopt [file pone.0009003.s005.tif]

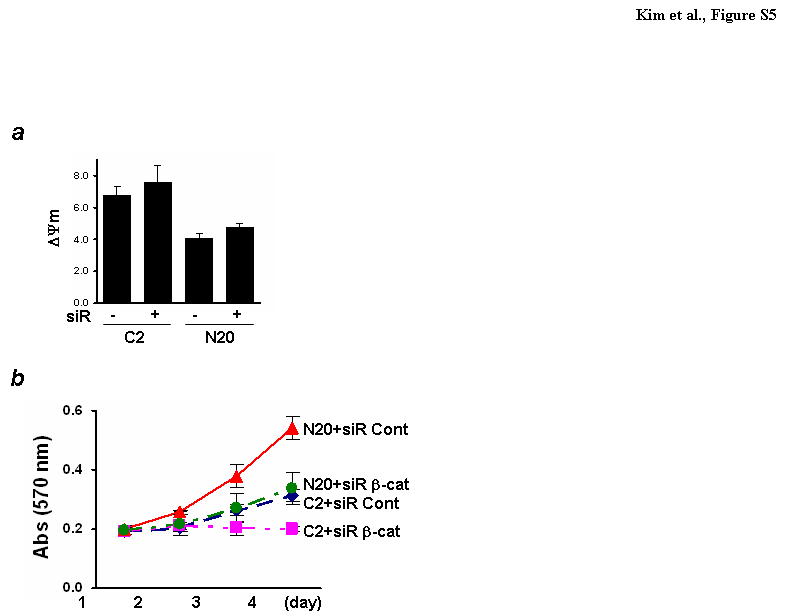

Supplement: Figure S5 — β-catenin knock-down inhibits cell growth. a, Δψm was determined after staining cells with a membrane potential sensitive dye, JC-1. Fluorescence intensity was acquired by reading cells in a fluorescence microplate reader. Values are expressed as means ± SD, and experiments were done in triplicate. b, Cellular growth was evaluated by the MTT cell growth assay for 4 days after transfection of β-catenin siRNA. No significant difference in cell growth was found in 24 hr, but cell growth was inhibited in both C2 and N20 cells by the β-catenin knockdown after 3 days of incubation. Data are expressed as absorbance at 570 nm and experiments were repeated twice in triplicate. (1.46 MB TIF) [file pone.0009003.s006.tif]

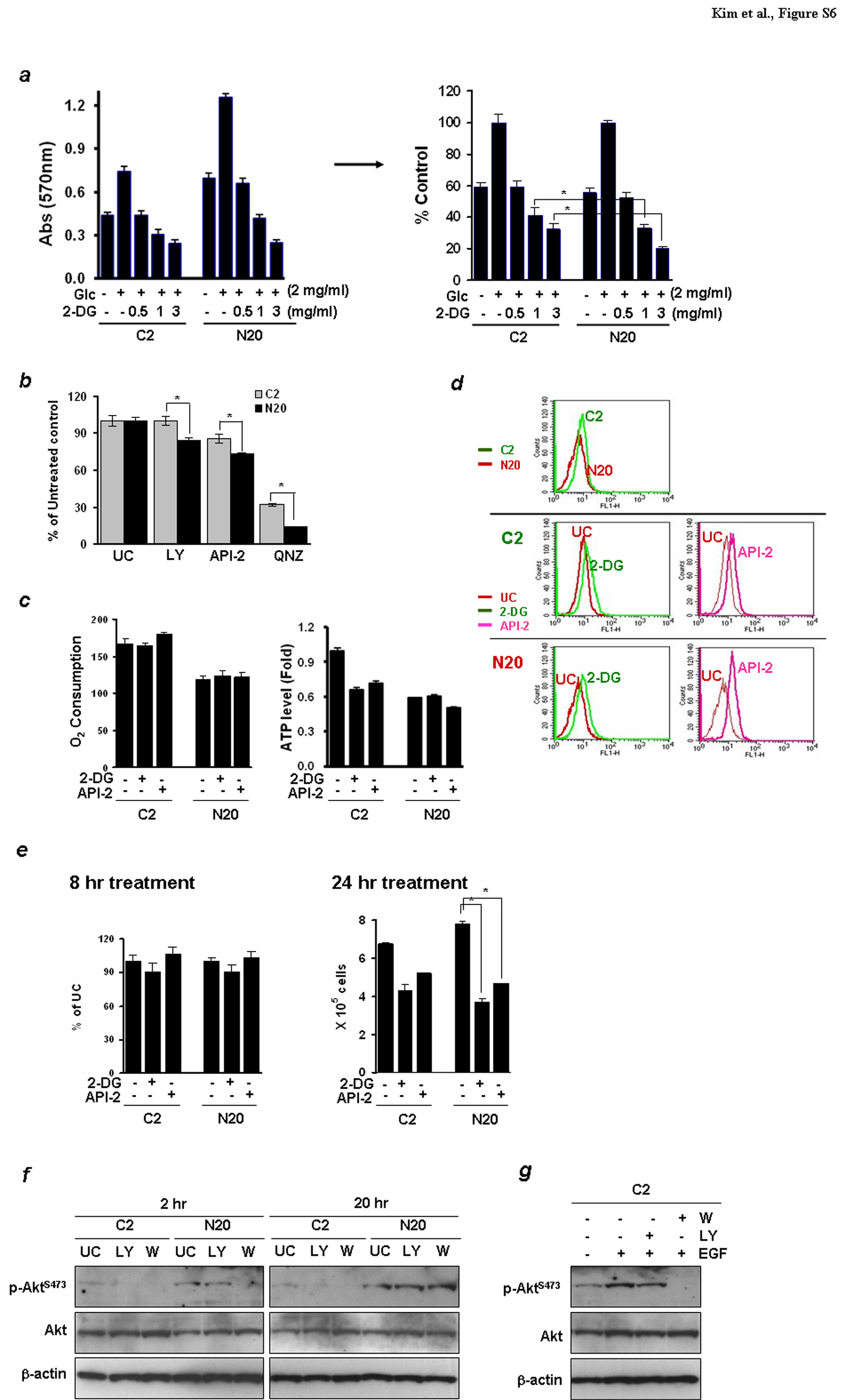

Supplement: Figure S6 — Cells with low expression of NEFH are more sensitive to API-2 or 2-DG. a, Cells were incubated in complete growth medium (2 mg/ml glucose) in the presence of 2-DG (0 {similar, tilde operator } 3 mg/ml) or were incubated under glucose-free condition for 24 hrs, and cell viability was assessed by the MTT assay. Values are expressed as means ± SD of absorbance at 570 nm (left), or % of control (no treatment) in each cell line. Experiments were repeated twice and done in triplicate. *, P<0.05. b, Cell viability was evaluated 24 hrs after the treatment of inhibitors. UC, untreated control; LY, LY294002 (10 µM); API-2 (10 µM); QNZ (50 µM). c, Oxygen consumption (left) and ATP levels (right) were measured after treatment of 2-DG or API-2. d, Representative results of ROS imaging after 2-DG or API-2 treatment in C2 and N20 cells. e, In 8 hrs, cell viability was not significantly decreased by 2-DG or API-2 treatment (left, MTT assay), but inhibition of cell viability was observed at 24 hrs of treatment (right, coulter counting). f, C2 and N20 cells were treated with LY294002 (10 µM) and Wortmannin (1 µM) for 2 and 20 hrs, and western blot analysis was performed. g, C2 cells were pre-treated with LY294002 and Wortmannin for 1 hr, and EGF (10 ng/ml) was added. Cells were then further incubated for 2 hrs, and western blot analysis was performed. Similar results were observed in cells treated with inhibitors for 16 hrs (data not shown). (10.30 MB TIF) [file pone.0009003.s007.tif]
